# Profiling *Thermus thermophilus* Argonaute Guide DNA Sequence Preferences by Functional Screening

**DOI:** 10.3389/fmolb.2021.670940

**Published:** 2021-04-29

**Authors:** Eric A. Hunt, Esta Tamanaha, Kevin Bonanno, Eric J. Cantor, Nathan A. Tanner

**Affiliations:** ^1^Applications and Product Development, New England Biolabs, Ipswich, MA, United States; ^2^Research, New England Biolabs, Ipswich, MA, United States

**Keywords:** prokaryotic argonaute, *Thermus thermophilus*, nucleic acid guide, targeted endonuclease, capillary electrophoresis

## Abstract

Prokaryotic Argonautes (pAgo) are an increasingly well-studied class of guided endonucleases, and the underlying mechanisms by which pAgo generate nucleic acid guides *in vivo* remains an important topic of investigation. Recent insights into these mechanisms for the Argonaute protein from *Thermus thermophilus* has drawn attention to global sequence and structural feature preferences involved in oligonucleotide guide selection. In this work, we approach the study of guide sequence preferences in *T. thermophilus* Argonaute from a functional perspective. Screening a library of 1,968 guides against randomized single- and double-stranded DNA substrates, endonuclease activity associated with each guide was quantified using high-throughput capillary electrophoresis, and localized sequence preferences were identified which can be used to improve guide design for molecular applications. The most notable preferences include: a strong cleavage enhancement from a first position dT independent of target sequence; a significant decrease in activity with dA at position 12; and an impact of GC dinucleotides at positions 10 and 11. While this method has been useful in characterizing unique preferences of *T. thermophilus* Argonaute and criteria for creating efficient guides, it could be expanded further to rapidly characterize more recent mesophilic variants reported in the literature and drive their utility toward molecular tools in biology and genome editing applications.

## Introduction

Prokaryotic Argonautes (pAgo) are programmable endonucleases involved in cellular defense against foreign genetic elements (Olovnikov et al., 2013; Swarts et al., 2014b, 2015; Koonin, 2017; Kuzmenko et al., 2020). While their eukaryotic homologs function as part of RNA silencing complexes and regulate mRNA, pAgo enzymes are functional as independent proteins and exhibit a wider range of activities with respect to both substrate and guide nucleic acid content (Hegge et al., 2018). *Thermus thermophilus* pAgo (TtAgo), profiled in detail in this and earlier studies (Sheng et al., 2014; Swarts et al., 2014a, 2017), uses short single-stranded DNA (ssDNA) guides to target DNA substrates. Other pAgos have been reported to use DNA guides to target RNA, or RNA guides or target either DNA or RNA (Yuan et al., 2005; Kaya et al., 2016; Miyoshi et al., 2016). Many studies of pAgo have drawn comparison to CRISPR-Cas systems and been proposed as alternative platforms for genome editing, though so far these aspirations have been premature as the functional underpinnings of pAgo are still being discovered (Lee et al., 2016; Hegge et al., 2018; Jolly et al., 2020). Recent reports of pAgo from mesophilic bacteria (García-Quintans et al., 2019; Hegge et al., 2019; Kuzmenko et al., 2019) have brought renewed excitement around pAgos as potential new molecular tools for genome editing. In addition to any possible *in vivo* utility, pAgos represent a novel class of nucleases for biotechnology, with targeting guided by simple DNA oligonucleotides (Enghiad and Zhao, 2017). While no specific sequence motifs such as the PAM sequence requirements of Cas proteins have been identified for pAgo, variability among targetable sequences has been observed (Swarts et al., 2014a; Enghiad and Zhao, 2017). Much of this variation has been attributed to global sequence attributes such as GC content or secondary structure (Swarts et al., 2017), but the bulk of knowledge concerning guide characteristics has been limited to sequencing pools of guides which are co-purified with pAgo (Olovnikov et al., 2013; Swarts et al., 2014a; Hegge et al., 2019; Kuzmenko et al., 2020). While these efforts have revealed much about the kinds of guides generated and associated with pAgo *in vivo*, they cannot directly identify sequence motifs that are inherently tied to successful substrate cleavage. Some studies have analyzed a small number of guide sequences *in vitro* to determine base content and positional effects, with evidence for TtAgo having a preference of a 5′dC for guides created *in vivo* (Swarts et al., 2014a, 2017), but the scale is still much smaller than the sequencing of co-purified, associated guides.

As pAgos are increasingly considered as potential tools for *in vitro* – and possibly *in vivo* – applications, understanding the preferences and behavior of pAgos with designed oligonucleotide guides will be essential for their successful utility and greater adoption. The eukaryotic Argonaute human AGO2 has been studied with high-throughput siRNA profiling (Becker et al., 2019), but to our knowledge there has been no systematic study of sequence preferences for pAgo or optimization of DNA guides for optimal TtAgo cleavage activity. In order to thoroughly characterize TtAgo DNA guide parameters, we devised a high-throughput method centered on capillary electrophoresis separation of fluorescently labeled oligonucleotides substrates (Greenough et al., 2016) to explore a large set of 1,968 guides which target randomized sequences over a range of GC contents. This methodology should have broad utility in rapidly characterizing new Argonautes, and may also be applied to the characterization of new CRISPR-Cas proteins and other classes of guided endonucleases.

## Materials and Methods

Recombinant TtAgo was expressed and purified from Escherichia *coli* according to the methods described in Hunt et al. (2018). Briefly, a synthetic, codon-optimized sequence with *N*-terminal hexa-histidine tag was expressed in T7 Express LysY/Iq *E. coli* (New England Biolabs, Inc.) and purified on an ÄKTA fast protein liquid chromatography system (GE Healthcare Life Sciences) using a 5 mL HisTrap FF column (GE Healthcare Life Sciences) for immobilized metal ion affinity chromatography. Purified protein was dialyzed into a storage buffer of 10 mM Tris–HCl, pH 7.4; 300 mM NaCl; 1 mM DTT; 0.1 mM EDTA; and 50% glycerol.

Guides were ordered in 96 well plate format (Integrated DNA Technologies, Inc.) and phosphorylated using T4 Polynucleotide Kinase (New England Biolabs, Inc.). Guide oligonucleotides were ordered from IDT in wet format with a normalized yield of 500 pmol suspended in 50 μL of nuclease-free water. The 5′ ends of the guides were phosphorylated by transferring 50 pmol of guide into a 50 μL reaction containing 10 U of T4 Polynucleotide Kinase (T4 PNK) in 50 mM Tris–HCl, pH 7.5; 10 mM MgCl_2_; 1 mM ATP; and 10 mM DTT. Reactions were incubated at 37°C for 1 h followed by a heat denaturation step at 65°C for 20 min. Guides were used directly without further purification.

Intact mass analysis was performed by Tandem Liquid Chromatography-Mass Spectrometry (LC-MS/MS) on an Vanquish Horizon UHPLC System equipped with a diode array detector and a Thermo Q-Exactive Plus mass spectrometer operating under negative electrospray ionization mode (–ESI). HPLC desalting and separation was performed using DIEA-HFIP ion-paring condition on a Thermo DNAPac RP column (2.1–50 mm) with the gradient mobile phase consisting of methanol and aqueous buffer. UV signal was recorded at 260 nm. MS data acquisition was performed in the scan mode (600 to 2500 *m/z*) at 70,000 mass resolution. ESI-MS raw data was deconvoluted using Promass HR (Novatia Inc.).

All substrates were obtained from Integrated DNA Technologies, Inc. and were received in a dried, HPLC-purified format. Upon receiving the substrates they were resuspended in nuclease-free water to a final concentration of 100 μM and stored at −20°C. Aliquots were prepared at a concentration of 200 nM in nuclease-free water for downstream use in reactions and were also stored at −20°C. Single- and double-substrates contained a 5′-FAM dye on the forward strand, and double-stranded substrates additionally contained a 5′-HEX dye on the reverse strand so TtAgo activity on both strands could be tracked by Capillary electrophoresis (CE) analysis (see Supplementary Material for full substrate sequences).

All reactions were carried out in polypropylene 96-well PCR plates (VWR International, LLC) sealed with Microseal^®^ B clear adhesive plate seals (Bio-Rad Laboratories, Inc.). Reactions with a final volume of 10 μL were setup in ThermoPol^®^ Reaction Buffer: 20 mM Tris–HCl, pH 8.8; 10 mM KCl; 10 mM ammonium sulfate; 2 mM MgSO_4_; and 0.1% Triton X-100 (New England Biolabs, Inc.). Each reaction contained 20 nmol TtAgo, 100 nmol 5′-phosphorylated guide oligo, and 20 nmol of fluorophore-labeled single- or double-stranded CE substrate. After all components were added at room temperature, plates were sealed and reactions were briefly mixed and centrifuged to collect the liquid in the bottom of the wells. Reactions were incubated at 75°C for 30 min in a T100^TM^ Thermal Cycler (Bio-Rad Laboratories, Inc.). Following incubation, plates were retrieved and centrifuged briefly to collect liquid in the bottom of the wells. Next, 40 μL of a 5 mU/μL dilution of Proteinase K (New England Biolabs, Inc.) in water was added to each well. The plates were once again sealed and incubated at 40°C for 20 min. CE analysis followed directly.

Capillary electrophoresis analysis was performed on an Applied Biosystems 3730xl DNA Analyzer with 36 cm capillary array and 5 s injection time. Peak assignment was performed with Peak Scanner^TM^ Software v1.0 (Thermo Fisher Scientific) using GeneScan^TM^ 120 LIZ^TM^ Dye Size Standard (Thermo Fisher Scientific) and the Local Southern method provided with the Peak Scanner^TM^ software.

Quantitative analysis and visualization of CE data was performed in the RStudio integrated development environment^1^ using the R programming language^2^ and associated packages from the Tidyverse^3^ including ggplot2^4^. Logo plots were created with the ggseqlogo package^5^, and some esthetic plot modifications were made with the lemon^6^ and cowplot^7^. Box plots are presented in the style of Tukey with population median represented by the center line and upper and lower quartiles represented by the boundaries of the box. Boxplots have been annotated for statistical significance between relevant groups using the following labels: ns (not significant) for *p* > 0.05; ^∗^ for *p* ≤ 0.05, ^∗∗^ for *p* ≤ 0.01; ^∗∗∗^ for *p* ≤ 0.001; and ^****^ for *p* ≤ 0.0001. *P*-values were determined using Kruskal–Wallis omnibus test for analysis of variance followed by *post hoc* Dunn’s test for multiple pairwise comparisons where *H*_0_ was rejected. *P*-values were adjusted for family-wise (Type 1) error using the Bonferroni method. Details of the statistical analysis, performed with the rstatix package^8^, including tables of *p*-values are provided in the Supplementary Material. Functions written in R by the authors have been made available as an open-source R package^9^. A full list of R packages used in this work, including those within the Tidyverse collection, may be found in the Supplementary Material.

## Results

In a previous work we utilized three double-stranded 98 nucleotide random sequences of different GC contents: 30, 48, and 67% GC (Hunt et al., 2018). We next sought to gain a greater understanding of Ago cleavage based on the sequence of the guide, and generated a set of fully-complementary guides for both forward and reverse strands of all substrates. The guides were generated by indexing a 17 nt window across the substrate sequences one base at a time, thus generating 82 guides per strand per substrate for a total of 492 fully-complementary guides. Additional guides were generated by altering the base identity of the first position of the 492 fully-complementary guides to the other corresponding bases, thus creating partially-complementary guides in which the 5′ end of the guide is not complementary to the 3′ end of the targeted sequence. Reactions were carried out in 96-well PCR plate format where one individual guide was mixed with TtAgo and the corresponding single- or double-stranded DNA substrate, and specific (as well as any off-target or non-specific) cleavage activity was measured by CE analysis of the resulting fluorescently-labeled product fragment size (Greenough et al., 2016). For convenience, a list of standard abbreviations used in this manuscript for nucleotide populations can be found in Table 1.

**TABLE 1:** Reference table for commonly used one letter abbreviations representing different nucleic acid populations.

	**Guanosine**	**Adenosine**	**Cytidine**	**Thymidine**
G	●			
A		●		
C			●	
T				●
W		●		●
S	●		●	
R	●	●		
Y			●	●
H		●	●	●
B	●		●	●
D	●	●		●
V	●	●	●	
N	●	●	●	●

### Intact Mass Analysis of T4 PNK Phosphorylated Guides

A subset of the guides utilized in this study were selected for intact mass analysis to determine if guide phosphorylation by T4 PNK could potentially skew the measured guided-endonuclease activity, as TtAgo requires guides to possess a 5′ phosphate moiety for loading. It has been reported in the literature that several factors can affect T4 PNK phosphorylation of DNA oligonucleotides (Van Houten et al., 1998). Houten et al. cite enzyme quality and oligo purity as two critical factors in phosphorylation outcome, and that even under the most optimal conditions used in that study T4 PNK phosphorylated an oligonucleotide with 5′dG more efficiently than one with 5′dA or 5′dC, and significantly more efficiently than one with 5′dT.

We have a high degree of confidence in the quality and purity of the commercially obtained oligonucleotides and T4 PNK used in this study. As such, a set of 16 guides before and after T4 PNK phosphorylation were analyzed by intact mass analysis. This guide subset was comprised of: (1) three sets of the same g2–g17 guide sequence with all combinations of g1, where measured activity levels varied from below 20% to above 80% depending on the identity of g1; and (2) four unique g1T sequences containing the least active g10/g11 dinucleotides (CC, CG, GC, and GG) with measured activity levels below 20%. This selection is representative of the most prominent conclusions drawn in this study which could be convoluted by incomplete phosphorylation. Of the 16 guides tested, all guides were phosphorylated to completion (compared to reaction controls without T4 PNK) with the exception of one guide, which contained unphosphorylated starting material at 1.3% abundance. A table of results are provided in the Supplementary Material along with the guide sequences selected for analysis.

All guided-endonuclease reactions in this experiment were carried out using a 5X molar excess of 5′-phosphorylated guide over TtAgo. This in conjunction with the mass analysis results lead us to the conclusion that T4 PNK phosphorylation efficiency did not skew the results in this study. With respect to the results obtained by Houten et al., we attribute the lack of variability in phosphorylation reactions carried out in this study to the way in which the reactions were setup. Houton et al. carried out phosphorylation with [Ɣ-^32^P]ATP, a far more expensive reagent, in equimolar ratio with oligonucleotide. As inorganic phosphate (P_i_) and pyrophosphate (PP_i_) are inhibitors of T4 PNK kinase activity, we carried out reactions with the far less expensive non-radioactive ATP in approximately 10^7^ molar excess of oligonucleotide, thus driving the reaction to completion.

### Activity Correlation With the First Base of the DNA Guide

Bases included in the guide and target sequences are referenced with a *g* or *t*, respectively, followed by a number representing position. The first position of the guide (g1) refers to the 5′ end, and the corresponding first position of the target (t1) refers to the 3′ end of the targeted sequence within the substrate.

#### Fully-Complementary Guides

Among the set of guides fully-complementary to the targeted sequence, low guided-endonuclease activity was observed on high-GC content double-stranded substrates. This was consistent with our previous study that demonstrated a requirement for single-stranded binding protein or helicase to achieve cleavage of high-GC dsDNA (Hunt et al., 2018). This inherent inefficiency does not appear to be addressable through guide sequence alone, and preference for lower GC content substrates has been observed elsewhere in the literature (Swarts et al., 2014a, 2017), thus it is likely a trait inherent to TtAgo, but not necessarily all pAgos.

**FIGURE 1:**
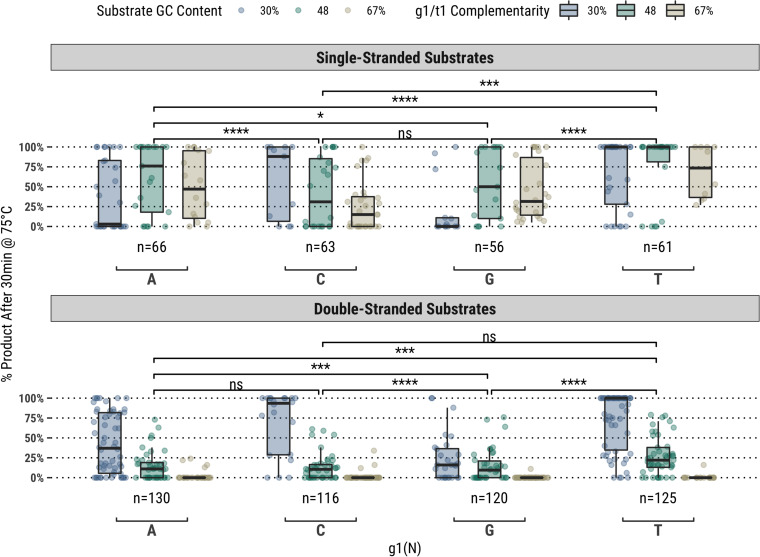
In a population of fully-complementary 17 nucleotide-long guides targeting randomized-sequence single- and double-stranded substrates of various GC contents, the first position of the guide (g1) was strongly correlated with overall guided-endonuclease activity of TtAgo. In particular, guides with g1T performed better than those with g1V. Each g1 grouping is divided to show the overall GC content of the substrate targeted, however, the significance levels shown are for each g1 grouping against the others. Boxplots have been annotated for statistical significance between relevant groups using the following labels: ns (not significant) for *p* > 0.05; ^∗^ for *p* ≤ 0.05; ^∗∗∗^ for *p* ≤ 0.001; and ^****^ for *p* ≤ 0.0001.

It was observed for the same fully-complementary set that guides starting with a thymidine (g1T) exhibited higher guided-endonuclease activity across all single- and double-stranded DNA substrates, though this subset of fully-complementary guides still performed poorly on the high-GC content double-stranded substrates (Figure 1). For the other bases (g1V), major differences in overall activity were observed for different GC content substrates, particularly on ssDNA substrates. For example, guides starting with cytidine (g1C) showed greater activity on the 30% GC content substrate compared to g1R, whereas those starting with adenosine (g1A) showed greater activity on the higher GC content substrates compared to g1S (Figure 1).

#### Partially-Complementary Guides

**FIGURE 2:**
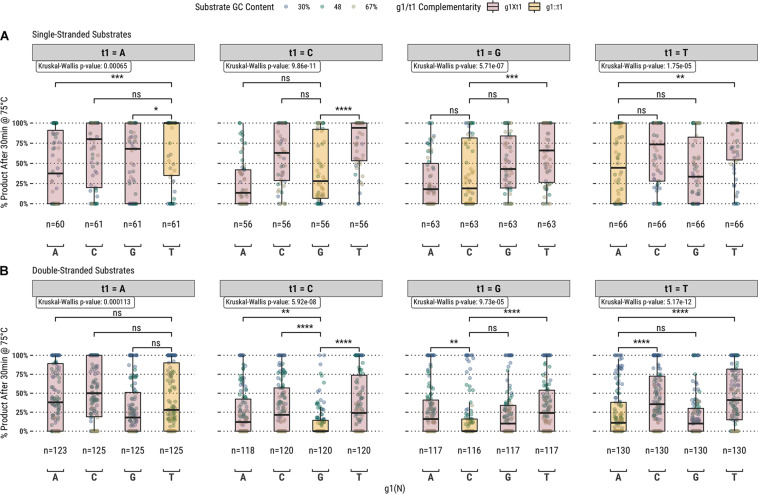
Guided-endonuclease activity of TtAgo using fully-complementary guides, where g1 is complementary to t1 (g1:t1), and partially complementary guides, where g1 is not complementary to t1 (g1Xt1) acting on **(A)** single-stranded and **(B)** double-stranded DNA substrates. Significance levels shown only for g1:t1 to g1Xt1 comparisons. Boxplots have been annotated for statistical significance between relevant groups using the following labels: ns (not significant) for *p* > 0.05; ^∗^ for *p* ≤ 0.05, ^∗∗^ for *p* ≤ 0.01; ^∗∗∗^ for *p* ≤ 0.001; and ^****^ for *p* ≤ 0.0001.

Higher guided-endonuclease activity associated with g1T in the fully-complementary set of guides highlighted the first position of the guide as a candidate for mutation. To further explore the overall effect of g1 on guide activity, we also screened a library of 1,476 guides targeting all the same sequences in the fully-complementary set, but with partial complementarity to the target sequence at g1; i.e., for the sliding 17 nucleotide window across each substrate, every sequence from bases 2–17 of the window was targeted by four separate guides containing either g1G, g1A, g1C, or g1T (Figure 2).

**FIGURE 3:**
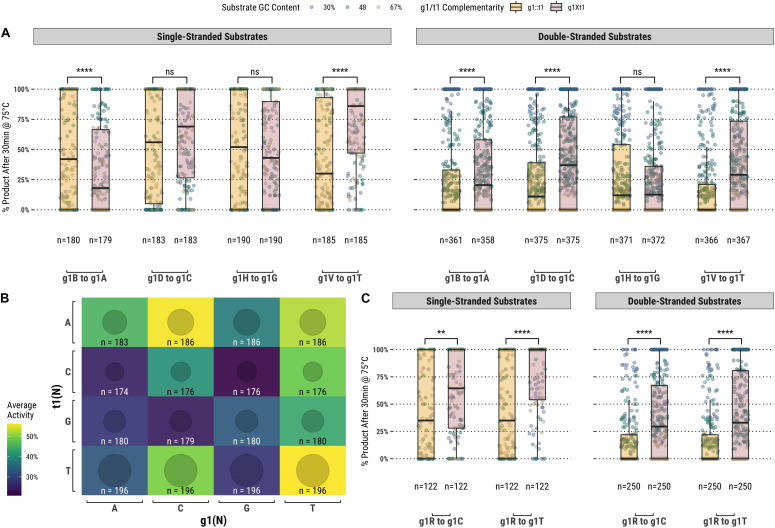
**(A)** Comparison of fully-complementary g1B/D/H/V guides to partially-complementary (g1 not complementary to t1) g1A/C/G/T guides. **(B)** A heatmap comparing the average activity of all g1-t1 combinations with single- and double-stranded substrates. **(C)** Comparing partially-complementary g1C and g1T guides to the same population of fully-complementary g1R guides. Boxplots have been annotated for statistical significance between relevant groups using the following labels: ns (not significant) for *p* > 0.05; ^∗∗^ for *p* ≤ 0.01; and ^****^ for *p* ≤ 0.0001.

Among the set of guides partially-complementary to the single-stranded (Figure 2A) and double-stranded (Figure 2B) targets at the first position of the guide, overall higher guided-endonuclease activity was observed for guides beginning with a pyrimidine (g1Y), specifically those beginning with g1T. For targets where the first position was a guanosine or cytidine (t1S), complementary guides exhibited lower guided-endonuclease activity. The alteration of g1 so that it was no longer complementary to t1 generally provided more favorable guided-endonuclease activity when the change was to g1Y (Figure 3A). This trend is observed for both single- and double-stranded DNA substrates when strictly comparing purine to pyrimidine swaps for g1 (Figure 3C). Inspecting g1-t1 pairs for all guides used on both single- and double-stranded substrates reveals the highest average activity for g1Y-t1W pairs (Figure 3B).

### Activity Correlation With the Twelfth Base of the DNA Guide

**FIGURE 4:**
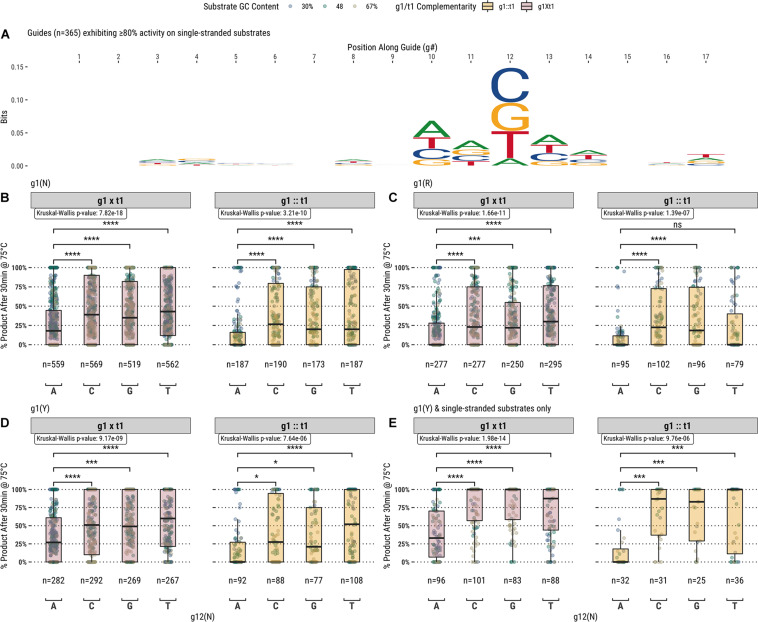
**(A)** Aligning guides which, when used with TtAgo, exhibit high activity (>80% product after 30 min at 75°C) highlights some sequence preferences for specific locations along the guide (g1 excluded from the alignment). **(B)** The preference for g12B is exemplified by the low activity of guides possessing g12A on single- and double-stranded substrates. **(C, D)** Comparing g1R and g1Y populations, the identity of g12 appears to be decoupled from the lower activity observed with the less favored g1R identity. **(E)** The effect of g12 on activity remains for g1Y and single-stranded substrates only, which are more readily accessible by TtAgo. Boxplots have been annotated for statistical significance between relevant groups using the following labels: ns (not significant) for *p* > 0.05; ^∗^ for *p* ≤ 0.05; ^∗∗∗^ for *p* ≤ 0.001; and ^****^ for *p* ≤ 0.0001.

In addition to the first position of the guide, analysis of the fully- and partially-complementary guide pool on single-stranded substrates identified other positions exhibiting potential sequence bias (Figure 4A). Among these positions, the most notable single location was position 12 of the guide (g12) which is immediately 3′ of the g10/g11 endonuclease cut site. Guides showing ≥80% activity with TtAgo on single-stranded substrates displayed a high frequency of a cytidine, guanosine, or thymidine at g12. To examine this further, TtAgo guided-endonuclease activity was plotted against g12 identity for single- and double-stranded DNA substrates (Figure 4B). Generally, guides with an adenosine at g12 performed poorly compared with those containing either cytidine, guanosine, or thymidine. The guide population was further restricted to examine the effect of g12 identity on the g1R (Figure 4C) and g1Y (Figures 4D,E) guide subsets.

### Activity Correlation With Guide Bases Surrounding the Cut-Site

**FIGURE 5:**
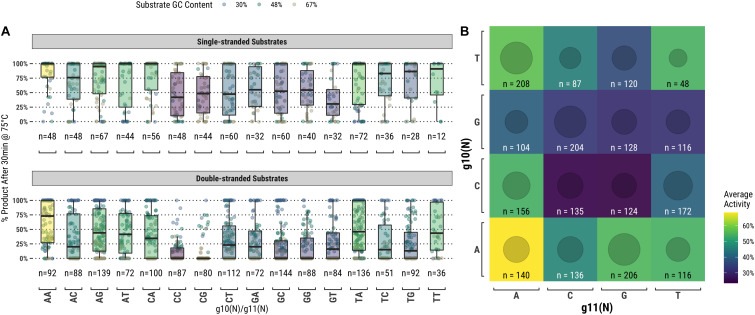
TtAgo shows a preference for low GC content dinucleotides at the g10/g11 positions which align with the enzyme active site. **(A)** Populations expressed as box plots individually for single- and double-stranded substrates. **(B)** Single- and double-stranded substrates combined and expressed as a heatmap.

TtAgo is known to cleave its substrate between the bases corresponding to the tenth and eleventh positions of the guide (g10 and g11, respectively). Figure 4A suggests that there may be some preference for sequence at these positions, but the relationship was not immediately clear from this analysis. We further examined these positions by measuring TtAgo guided-endonuclease activity against the g10/g11 dinucleotide identity (Figure 5). In general, it appeared that high GC content dinucleotides at the g10/g11 positions did not support efficient TtAgo activity. A guanosine or cytidine in g10 had a negative effect on overall activity, with the exception of the g10C/g11A dinucleotide. By far g10A dinucleotides performed the best, with g10A/g11A being the most active combination.

### Activity Correlation With Global Attributes

**FIGURE 6:**
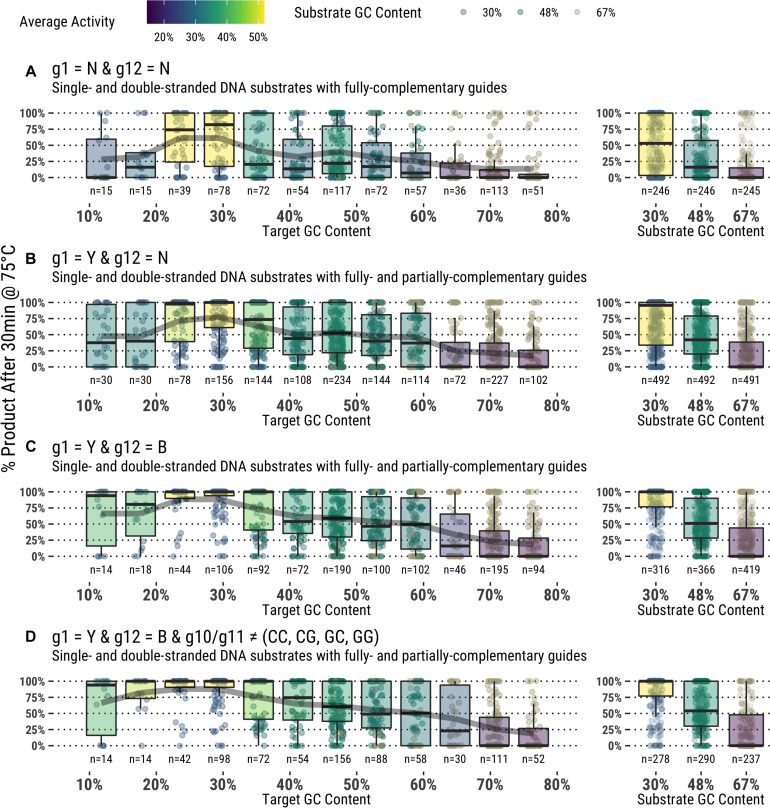
**(A–D)** Application of guide design principles to achieve highly active guides for *in vitro* use.

Attributes of the entire substrate and targeted sequence therein play a role in determining effective guided-endonuclease activity. Even under the most favorable conditions as determined from the data derived in this study, there is still a preference for TtAgo guided-endonuclease activity on lower GC content substrates and target sequences (Figure 6).

## Discussion

The most prominent guide sequence motif affecting overall guided-endonuclease activity of TtAgo was the identity of the nucleotide at g1. The strong influence of the g1 base alone is likely connected to the “chopping” mechanism described by Swarts et al. (2017), in which the first position of the target (t1) – located at the 3′ end of the target sequence corresponding to the complementary g1 5′ end of the guide – exhibits some level of interaction with the PIWI domain of TtAgo. Those findings indicated t1G had the most stable PIWI interactions and in this way influenced the observed skew of *in vivo* apo-TtAgo generated guides toward g1C (Swarts et al., 2017). Swarts et al., 2017 also investigated the number of hydrogen bonds which occur between the MID domain of TtAgo and g1N of the guide. While those findings established likely binding energies for g1N to be g1C ≅ g1T > g1A > g1G, the general conclusion was that g1N does not significantly affect guided-endonuclease activity of TtAgo.

While it is true that TtAgo can readily utilize guides starting with any base, g1N, our results suggested that there is preferentially higher activity associated with guides starting with a pyrimidine (g1Y), especially guides starting with g1T (Figure 3C). When comparing across the different g1N populations of guides, those starting with g1T (and also g1C to a lesser degree) improved the guided-endonuclease activity of the majority of guides in the population targeting t1B (or t1H in the case of g1C; Figure 3A). Apart from g1T being slightly preferred to g1C (Figures 3B,C), these results pertaining to TtAgo guided-endonuclease activity correlate well with the binding energy predictions made by Swarts et al. (2017), and it seems likely that these two aspects of TtAgo guide oligonucleotide function are correlated (Swarts et al., 2017).

Interestingly in the case of t1S, guides which were complementary at g1 exhibited lower guided-endonuclease activity (Figure 2). This provides some evidence for the suspicion of Swarts et al. (2017), that despite the lack of base pairing observed between g1 and t1 in TtAgo crystal structures, the possibility of base pairing between the two cannot be ruled out and could be one component which contributed to the extended binding dwell time they observed between the g1C and t1G guide-target pair (Swarts et al., 2017). The potential for interaction between g1 and t1 could reduce the observed endonuclease activity with these guides by slowing release following cleavage or disrupting the cleavage-competent conformation of TtAgo once the Argonaute nucleoprotein complex is bound to the target.

Another interesting observation was made regarding the bases surrounding the endonuclease active site of TtAgo, g10, and g11. GC-rich g10/g11 dinucleotides were associated with lower overall guided-endonuclease activity for TtAgo. Perhaps the proclivity of TtAgo for low GC t1-g1 pairs and g10/g11 dinucleotides is related to a mechanism of self-discrimination in *T. thermophilus* (Rocha and Danchin, 2002). The organism has a very high genomic GC content ≅69% (Jiang et al., 2013). A t1S-g1S pair or high GC g10/g11 dinucleotide in a guide would have a higher probability of having been derived from the host rather than an invasive genetic element with lower GC content.

Each of these observations about specific guide characteristics can inform study of TtAgo preferences, and looking at the various parameters together can give a complete picture of guide design and performance. Figure 6 combines each factor described above to select for only guides fitting the suggested criteria for higher TtAgo activity, and indeed an overall improvement is seen with guides that: (1) have a thymidine or cytidine at g1 regardless of target base; (2) avoid adenosine at g12; and (3) avoid g10S/g11S dinucleotides. Despite a notable improvement in overall activity, many guides meeting these conditions still displayed poor cleavage, particularly with high GC content and dsDNA. We were unable to determine any specific positions or sequences with particularly poor performance other than those described above and general preference for 30–50% GC targets. Further study will aim to better understand more nuanced guide preferences and activities, as well as potential effects of buffer additives and formulation on TtAgo and other guided-endonuclease activity with a similar study design.

While the method presented herein is well adapted for high-throughput analysis of pAgo guide sequences, it is not without limitations. It should be noted that the guide sequence preferences observed in this study are specific to TtAgo and are not indicative of guide sequence preferences that may be inherent to other pAgos. This study utilized a single set of reaction conditions for all guided-endonuclease reactions, and while the conditions were selected to be optimal for TtAgo activity, different reaction buffer or temperature conditions could have an effect on the observed guide sequence preferences. Furthermore, it should also be noted that a result of low guided-endonuclease activity in this manuscript cannot conclusively assign the absence of activity to a specific sequence motif (e.g., high GC content dinucleotides g10S/g11S), but rather is demonstrative of a slower reaction rate associated with these motifs, as the duration of all reaction incubations was fixed.

Additionally, because the guide sequences were selected by indexing a window across the substrate sequences, some sequence bias exists at the extremities of GC content distribution. This can be observed in the sample sizes comprising the population distributions represented by each box plot in Figure 1. Guides starting with g1W are over represented in the 30% GC content substrate and underrepresented in the 67% GC content substrate. This bias can be somewhat eliminated by binning the points according to the GC content of the targeted sequence rather than the entire substrate (Supplementary Figure 1), however, this binning can also create relatively small sample sizes that might reduce the reliability of the population distribution at each target sequence GC content bin. Nonetheless, this variability in activity at the extremities of the distribution is still an interesting observation, especially in consideration of previous publications highlighting the preference of TtAgo for g1C guides obtained *in vivo* for targeting low GC content sequences (Swarts et al., 2014a, 2017) in its capacity of host cellular defense against invading nucleic acid elements. The possibility of positional bias related to the extreme ends of substrates was also considered, and while some bias is observed for guides targeting the ends of high GC double-stranded substrates, the same bias is not observed in single-stranded substrate reactions. This was expected to some degree as it was previously observed double-stranded substrates are not readily accessible to TtAgo without the help of additional proteins (Hunt et al., 2018).

In conclusion, we used a high-throughput capillary electrophoresis method to screen a large population of ssDNA guides with TtAgo for guided-endonuclease activity against randomized sequence substrates of varying GC content. Our results generally agree with previous findings from *in vivo* generated guide pools co-purified with TtAgo, but by characterizing in detail a large number of guide sequences we were able to further demonstrate that optimal guide design for *in vitro* use of TtAgo does not mirror conditions which would be produced through an *in vivo* apo-TtAgo activity. Namely, using a pyrimidine in the first position of the guide yields higher overall activity even when this base is not complementary to the first base of the target, not expected given the current hypotheses regarding *in vivo* guide production by TtAgo. Additionally, we find that the twelfth position of the guide also play an important role in overall guide activity, and guides containing cytidine, guanosine, or thymidine in this position are strongly preferred over adenosine. An overall preference for moderate GC content in both single- and double-strand targets, with best performance observed for 30–50% GC sequences even with optimal g1 and g12 positions. Additionally, we observed a strong effect of GC content at positions g10 and g11 with g10W/g11W dinucleotides strongly preferred. As more pAgo are discovered and described in the literature, the high-throughput method described herein will serve as a useful tool for the rapid characterization of guide preferences between Argonautes derived from different species, and will help to advance this class of proteins toward broader utility as novel tools for molecular biology applications.

## Data Availability Statement

The original contributions presented in the study are included in the article/Supplementary Material, further inquiries can be directed to the corresponding author/s.

## Author Contributions

EH designed and coordinated experiments, analyzed data, and wrote the manuscript. KB, ET, and EH performed guided-endonuclease experiments with TtAgo to generate the capillary electrophoresis data used in this study. NT and EC oversaw the study and preparation of the manuscript. All authors contributed to the article and approved the submitted version.

## Conflict of Interest

At the time of this work, the authors were employed by New England Biolabs, Inc., which funded this work and is the manufacturer of reagents used in this manuscript, including *Thermus thermophilus* Argonaute. Even though the authors were employed and funded by New England Biolabs, Inc., this did not detract from the objectivity of data generation or its interpretation.

## Footnotes

1 https://rstudio.com/products/rstudio/

2 https://www.R-project.org/

3 https://www.tidyverse.org/

4 https://ggplot2.tidyverse.org

5 https://github.com/omarwagih/ggseqlogo

6 https://github.com/stefanedwards/lemon

7 https://github.com/wilkelab/cowplot

8 https://CRAN.R-project.org/package=rstatix

9 https://github.com/eric-hunt/fragr

## Supplementary Material

Supplementary Data Sheet 1 Supplementary figures, substrate sequences, and details for intact mass analysis and statistical treatment of data.

Supplementary Data Sheet 2 Capillary electrophoresis data for endonuclease reactions performed for this manuscript. Peak assignment performed with Peak Scanner^ < *c**p**s*:*s**c* > *T**M* < /*c**p**s*:*s**c* >^ v1.0 for blue and green channels from Applied Biosystems 3730xl DNA Analyzer FSA file output.
