# Supplementary material for: Profiling Thermus thermophilus Argonaute Guide DNA Sequence Preferences by Functional Screening
Source: Front Mol Biosci. 2021 Apr 29;8:670940. doi: 10.3389/fmolb.2021.670940 (PMC8118625; doi:10.3389/fmolb.2021.670940)
Supplement: Supplementary Data Sheet 1 — Supplementary figures, substrate sequences, and details for intact mass analysis and statistical treatment of data. [file Data_Sheet_1.PDF]

Supplementary Material

1 Supplementary Data

1.1 Raw Data Files

A data file in comma separated values (.csv) format is provided containing capillary electrophoresis data for the FAM (blue) and VIC (green) channels from the Applied Biosystems 3730xl DNA Analyzer used in this study. The file contains peaks called by the Peak Scanner™ software (Thermo Fisher Scientific) using the Local Southern method with reference to the GeneScan™ 120 LIZ™ Dye Size Standard (Thermo Fisher Scientific).

1.2 Sequences

Sequences for the fluorescently-tagged substrates obtained from Integrated DNA Technologies and used in this study are provided:

1.2.1 ssDNA Substrates

FAM-RAN30_98FW

```
/56-FAM/
ATCATATTGGAGTTGAATGTAGGTTATAATAATATTTACTAAGCGACTAAGTGCTCAGATAATATAACTGTAGCTTAAGCTTATTTACCC
TACACTAT
```

FAM-RAN48_98FW

```
/56-FAM/
CTCTACATTCTGGTGCAGCCCGATAAAGCCTACTGAGGGTTAAATAGCTAGTGCTCAGATCAGTCGTATGTAGGCTTGTGCACACCATTA
GCGGTAGC
```

FAM-RAN67_98FW

```
/56-FAM/
AACACCAGGGAGCTCCTGCGCTTGCCTAACGGGATATGCGCGACCGGCCAGTGCTCAGGGCACGCGACTGGTGGTCCGGCCAGCCGTCGT
TGCTGGTC
```

1.2.2 dsDNA Substrates

FW5FAM-RV5HEX_RAN30

```
/56-FAM/
ATCATATTGGAGTTGAATGTAGGTTATAATAATATTTACTAAGCGACTAAGTGCTCAGATAATATAACTGTAGCTTAAGCTTATTTACCC
TACACTAT
```

```
/5HEX/
ATAGTGTAGGGTAAATAAGCTTAAGCTACAGTTATATTATCTGAGCACTTAGTCGCTTAGTAAATATTATTATAACCTACATTCAACTCC
AATATGAT
```

FW5FAM-RV5HEX_RAN48

/56-FAM/
CTCTACATTCTGGTGCAGCCCGATAAAGCCTACTGAGGGTTAAATAGCTAGTGCTCAGATCAGTCGTATGTAGGCTTGTGCACACCATTA
GCGGTAGT

/5HEX/
ACTACCGCTAATGGTGTGCACAAGCCTACATACGACTGATCTGAGCACTAGCTATTTAACCCTCAGTAGGCTTTATCGGGCTGCACCAGA
ATGTAGAG

FW5FAM-RV5HEX_RAN67

/56-FAM/
AACACCAGGGAGCTCCTGCGCTTGCCTAACGGGATATGCGCGACCGGCCAGTGCTCAGGGCACGCGACTGGTGGTCCGGCCAGCCGTCGT
TGCTGGCT

/5HEX/
AGCCAGCAACGACGGCTGGCCGGACCACCAGTCGCGTGCCCTGAGCACTGGCCGGTCGCGCATATCCCGTTAGGCAAGCGCAGGAGCTCC
CTGGTGTT

1.3 Open-Source Software

A list of computational software packages used to analyze capillary electrophoresis data and generate all visualizations in this study is provided:

Peak Scanner™ (https://www.thermofisher.com/us/en/home/life-science/sequencing/fragment-analysis/fragment-analysis-fundamentals/fragment-analysis-software-data-analysis.html)

RStudio IDE (https://rstudio.com/products/rstudio/)

R programming language (https://www.R-project.org/)

tidyverse (https://www.tidyverse.org/)

- readr (https://readr.tidyverse.org/)
- tibble (https://tibble.tidyverse.org/)
- tidyr (https://tidyr.tidyverse.org/)
- dplyr (https://dplyr.tidyverse.org/)
- stringr (https://stringr.tidyverse.org/)
- magrittr (https://magrittr.tidyverse.org/)
- purrr (https://purrr.tidyverse.org/)
- ggplot2 (https://ggplot2.tidyverse.org/)
- glue (https://glue.tidyverse.org/)

rlang (https://rlang.r-lib.org/)

broom (https://broom.tidymodels.org/)

scales (https://scales.r-lib.org/)

ggseqlogo (https://github.com/omarwagih/ggseqlogo)

cowplot (https://github.com/wilkelab/cowplot)

lemon (https://github.com/stefanedwards/lemon)

rstatix (https://CRAN.R-project.org/package=rstatix)

fragr (https://github.com/eric-hunt/fragr)

1.4 Intact Mass Analysis Results

1.4.1 Control samples (pre T4 PNK treatment)

Ctrl. = control, oligo with reaction buffer, no enzyme, 37°C/1hr followed by 65°C/20min

Rxn. = reaction, oligo with reaction buffer and enzyme, 37°C/1hr followed by 65°C/20min

+Phos = the calculated weight of the phosphorylated oligo of same sequence as listed above

N.D. = not detected

			Monoisotopic MS		
	Ctrl./Rxn.	*Sequence*	*Calculated*	*Experimental*	*Abundance*
EH-1	Ctrl.	TTATTATAACCTACATT	5115.8973	5115.892	
		+Phos	5195.8637	N.D.	
EH-2	Ctrl.	TAGTCGCTTAGTAAATA	5205.9164	5205.912	
		+Phos	5285.8828	N.D.	
EH-3	Ctrl.	ATAAGCTTAAGCTACAG	5199.9283	5199.924	
		+Phos	5279.8947	N.D.	
EH-4	Ctrl.	TACATACGACTGATCTG	5166.9055	5166.901	
		+Phos	5246.8719	N.D.	
EH-5	Ctrl.	TAATGGTGTGCACAAGC	5231.9182	5231.914	
		+Phos	5311.8845	N.D.	
EH-6	Ctrl.	TTAAGCTTAAGCTACAG	5190.9168	5190.914	
		+Phos	5270.8831	5372.943	
EH-7	Ctrl.	TGGCTGGCCGGACCACC	5169.8912	5169.89	
		+Phos	5249.8576	N.D.	
EH-8	Ctrl.	TGCCAGCAACGACGGCT	5177.9076	5177.901	
		+Phos	5257.8739	N.D.	
EH-9	Ctrl.	AAGTCGCTTAGTAAATA	5214.928	5214.925	
		+Phos	5294.8943	N.D.	
EH-10	Ctrl.	AACATACGACTGATCTG	5175.9171	5175.915	
		+Phos	5255.8834	N.D.	
EH-11	Ctrl.	GAGTCGCTTAGTAAATA	5230.9229	5230.92	
		+Phos	5310.8892	N.D.	
EH-12	Ctrl.	GTAAGCTTAAGCTACAG	5215.9232	5215.921	
		+Phos	5295.8896	N.D.	
EH-13	Ctrl.	GACATACGACTGATCTG	5191.912	5191.912	
		+Phos	5271.8783	N.D.	
EH-14	Ctrl.	CAGTCGCTTAGTAAATA	5190.9168	5190.914	
		+Phos	5270.8831	N.D.	
EH-15	Ctrl.	CTAAGCTTAAGCTACAG	5175.9171	5175.915	
		+Phos	5255.8834	N.D.	
EH-16	Ctrl.	CACATACGACTGATCTG	5151.9059	5151.902	
		+Phos	5231.8722	N.D.	

1.4.2 Reaction samples (post T4 PNK treatment)

	Ctrl./Rxn.	Sequence	Monoisotopic MS Calculated	Experimental	Abundance
EH-17	Rxn.	TTATTATAACCTACATT	5115.8973	N.D.	
		+Phos	5195.8637	5195.861	
EH-18	Rxn.	TAGTCGCTTAGTAAATA	5205.9164	5205.9	1.30%
		+Phos	5285.8828	5285.882	
EH-19	Rxn.	ATAAGCTTAAGCTACAG	5199.9283	N.D.	
		+Phos	5279.8947	5279.892	
EH-20	Rxn.	TACATACGACTGATCTG	5166.9055	N.D.	
		+Phos	5246.8719	5246.87	
EH-21	Rxn.	TAATGGTGTGCACAAGC	5231.9182	N.D.	
		+Phos	5311.8845	5311.88	
EH-22	Rxn.	TTAAGCTTAAGCTACAG	5190.9168	N.D.	
		+Phos	5270.8831	5270.88	
EH-23	Rxn.	TGGCTGGCCGGACCACC	5169.8912	N.D.	
		+Phos	5249.8576	5249.861	
EH-24	Rxn.	TGCCAGCAACGACGGCT	5177.9076	N.D.	
		+Phos	5257.8739	5257.872	
EH-25	Rxn.	AAGTCGCTTAGTAAATA	5214.928	N.D.	
		+Phos	5294.8943	5294.893	
EH-26	Rxn.	AACATACGACTGATCTG	5175.9171	N.D.	
		+Phos	5255.8834	5255.88	
EH-27	Rxn.	GAGTCGCTTAGTAAATA	5230.9229	N.D.	
		+Phos	5310.8892	5310.888	
EH-28	Rxn.	GTAAGCTTAAGCTACAG	5215.9232	N.D.	
		+Phos	5295.8896	5285.889	
EH-29	Rxn.	GACATACGACTGATCTG	5191.912	N.D.	
		+Phos	5271.8783	5271.876	
EH-30	Rxn.	CAGTCGCTTAGTAAATA	5190.9168	N.D.	
		+Phos	5270.8831	5270.882	
EH-31	Rxn.	CTAAGCTTAAGCTACAG	5175.9171	N.D.	
		+Phos	5255.8834	5255.883	
EH-32	Rxn.	CACATACGACTGATCTG	5151.9059	N.D.	
		+Phos	5231.8722	5231.87	

1.4.3 Deconvoluted peak reports

[<<] [Top] [ESI Mass Spectrum] [Deconvolution] [View Data] [Log File]

Deconvolution Peak Report:

Mass (Da)	Intensity	Std. Dev.	Score	Delta Mass	%Relative	%Total
5115.892	4.97E+008	0.051	8.70	0.000	100.00	84.80
5429.946	2.85E+007	0.054	7.46	314.054	5.75	4.87
5137.867	2.78E+007	0.052	7.44	21.975	5.59	4.74
5116.892	2.07E+007	0.051	7.32	1.000	4.17	3.53
4811.840	6.41E+006	0.049	6.81	-304.052	1.29	1.09
5431.945	5.61E+006	0.054	6.75	316.053	1.13	0.96

ZNova 3.0.12 ©2001-2018 Novatia, LLC

[<<] [Top] [ESI Mass Spectrum] [Deconvolution] [View Data] [Log File]

Deconvolution Peak Report:

Mass (Da)	Intensity	Std. Dev.	Score	Delta Mass	%Relative	%Total
5205.912	2.84E+008	0.052	8.45	0.000	100.00	80.36
5227.881	3.13E+007	0.052	7.50	21.969	11.01	8.85
5519.962	1.30E+007	0.055	7.11	314.050	4.58	3.68
5206.913	9.30E+006	0.052	6.97	1.001	3.27	2.63
5228.882	7.66E+006	0.052	6.88	22.970	2.70	2.17
5520.962	5.26E+006	0.055	6.72	315.050	1.85	1.49
5249.858	2.91E+006	0.053	6.46	43.946	1.02	0.82

ZNova 3.0.12 ©2001-2018 Novatia, LLC

[<<] [Top] [ESI Mass Spectrum] [Deconvolution] [View Data] [Log File]

Deconvolution Peak Report:

Mass (Da)	Intensity	Std. Dev.	Score	Delta Mass	%Relative	%Total
5199.924	9.79E+006	0.052	6.99	0.000	100.00	80.50
5221.896	1.09E+006	0.052	6.04	21.972	11.15	8.98
5205.927	3.53E+005	0.052	5.55	6.003	3.61	2.90
5513.972	3.34E+005	0.055	5.52	314.048	3.41	2.75
5222.895	1.82E+005	0.052	5.26	22.971	1.86	1.50
5115.892	1.75E+005	0.051	5.24	-84.032	1.79	1.44
5514.973	1.30E+005	0.055	5.11	315.049	1.33	1.07
5200.928	1.05E+005	0.053	5.02	1.004	1.07	0.86

ZNova 3.0.12 ©2001-2018 Novatia, LLC

[<<] [Top] [ESI Mass Spectrum] [Deconvolution] [View Data] [Log File]

Deconvolution Peak Report:

Mass (Da)	Intensity	Std. Dev.	Score	Delta Mass	%Relative	%Total
5166.901	1.14E+008	0.052	8.06	0.000	100.00	84.64
5188.874	1.04E+007	0.053	7.02	21.973	9.13	7.73
5480.955	4.39E+006	0.055	6.64	314.054	3.86	3.26
5167.904	2.53E+006	0.052	6.40	1.003	2.22	1.88
5189.865	1.96E+006	0.054	6.29	22.964	1.72	1.46
5184.907	1.38E+006	0.052	6.14	18.006	1.21	1.03

ZNova 3.0.12 ©2001-2018 Novatia, LLC

[<<] [Top] [ESI Mass Spectrum] [Deconvolution] [View Data] [Log File]

Deconvolution Peak Report:

Mass (Da)	Intensity	Std. Dev.	Score	Delta Mass	%Relative	%Total
5231.914	1.97E+007	0.052	7.29	0.000	100.00	78.80
5253.885	2.58E+006	0.052	6.41	21.971	13.13	10.35
5166.901	1.23E+006	0.052	6.09	-65.013	6.27	4.94
5545.965	6.76E+005	0.055	5.83	314.051	3.44	2.71
5254.885	5.94E+005	0.053	5.77	22.971	3.02	2.38
5546.964	2.07E+005	0.055	5.32	315.050	1.05	0.83

ZNova 3.0.12 ©2001-2018 Novatia, LLC

[<<] [Top] [ESI Mass Spectrum] [Deconvolution] [View Data] [Log File]

Deconvolution Peak Report:

Mass (Da)	Intensity	Std. Dev.	Score	Delta Mass	%Relative	%Total
5372.943	8.49E+006	0.054	6.93	0.000	100.00	36.45
5190.914	3.54E+006	0.052	6.55	-182.029	41.72	15.20
5246.904	2.24E+006	0.052	6.35	-126.039	26.39	9.62
5328.949	2.12E+006	0.053	6.33	-43.994	24.93	9.09
5400.916	1.33E+006	0.054	6.12	27.973	15.65	5.70
5394.917	9.17E+005	0.055	5.96	21.974	10.80	3.94
5166.903	6.08E+005	0.052	5.78	-206.040	7.16	2.61
5686.995	5.07E+005	0.057	5.71	314.052	5.97	2.18
5327.943	3.99E+005	0.055	5.60	-45.000	4.69	1.71
5373.944	2.12E+005	0.054	5.33	1.001	2.49	0.91
5687.996	2.06E+005	0.057	5.31	315.053	2.42	0.88
5428.926	2.04E+005	0.054	5.31	55.983	2.40	0.87
5189.897	1.79E+005	0.056	5.25	-183.046	2.11	0.77
5350.932	1.79E+005	0.055	5.25	-22.011	2.10	0.77
5329.956	1.72E+005	0.053	5.23	-42.987	2.02	0.74
5282.928	1.68E+005	0.054	5.22	-90.015	1.97	0.72
5231.911	1.59E+005	0.054	5.20	-141.032	1.88	0.68
5401.912	1.47E+005	0.055	5.17	28.969	1.73	0.63
5212.882	1.45E+005	0.052	5.16	-160.061	1.71	0.62
5326.937	1.44E+005	0.055	5.16	-46.006	1.69	0.62
5642.993	1.43E+005	0.056	5.16	270.050	1.68	0.61
5278.929	1.34E+005	0.053	5.13	-94.014	1.58	0.58
5205.924	1.34E+005	0.052	5.13	-167.019	1.58	0.57
4941.832	1.26E+005	0.054	5.10	-431.111	1.48	0.54
5422.897	1.11E+005	0.054	5.04	49.954	1.30	0.47
5504.965	1.11E+005	0.055	5.04	132.022	1.30	0.47
5395.917	1.07E+005	0.054	5.03	22.974	1.26	0.46
5708.971	9.61E+004	0.057	4.98	336.028	1.13	0.41
5505.962	9.41E+004	0.055	4.97	133.019	1.11	0.40
5416.896	8.99E+004	0.054	4.95	43.953	1.06	0.39
5493.957	8.88E+004	0.061	4.95	121.014	1.05	0.38

ZNova 3.0.12 ©2001-2018 Novatia, LLC

[<<] [Top] [ESI Mass Spectrum] [Deconvolution] [View Data] [Log File]

Deconvolution Peak Report:

Mass (Da)	Intensity	Std. Dev.	Score	Delta Mass	%Relative	%Total
5169.890	3.30E+006	0.052	6.52	0.000	100.00	67.90
5191.860	4.33E+005	0.052	5.64	21.970	13.14	8.92
5166.898	2.68E+005	0.052	5.43	-2.992	8.14	5.53
5168.903	2.66E+005	0.052	5.43	-0.987	8.07	5.48
5190.895	1.60E+005	0.052	5.20	21.005	4.84	3.28
5225.879	1.12E+005	0.054	5.05	55.989	3.40	2.31
5483.944	1.05E+005	0.055	5.02	314.054	3.20	2.17
5192.871	5.80E+004	0.052	4.76	22.981	1.76	1.19
5188.892	4.20E+004	0.052	4.62	19.002	1.27	0.86
5226.881	4.09E+004	0.052	4.61	56.991	1.24	0.84
5505.898	4.00E+004	0.055	4.60	336.008	1.21	0.82
5231.918	3.32E+004	0.060	4.52	62.028	1.01	0.68

ZNova 3.0.12 ©2001-2018 Novatia, LLC

[<<] [Top] [ESI Mass Spectrum] [Deconvolution] [View Data] [Log File]

Deconvolution Peak Report:

Mass (Da)	Intensity	Std. Dev.	Score	Delta Mass	%Relative	%Total
5177.901	6.01E+006	0.052	6.78	0.000	100.00	74.55
5199.877	1.10E+006	0.052	6.04	21.976	18.32	13.66
5200.870	2.77E+005	0.057	5.44	22.969	4.61	3.43
5166.901	2.09E+005	0.052	5.32	-11.000	3.47	2.59
5491.952	1.79E+005	0.055	5.25	314.051	2.98	2.22
4953.824	1.57E+005	0.050	5.20	-224.077	2.62	1.95
5221.845	6.47E+004	0.053	4.81	43.944	1.08	0.80
5513.929	6.44E+004	0.055	4.81	336.028	1.07	0.80

ZNova 3.0.12 ©2001-2018 Novatia, LLC

[<<] [Top] [ESI Mass Spectrum] [Deconvolution] [View Data] [Log File]

Deconvolution Peak Report:

Mass (Da)	Intensity	Std. Dev.	Score	Delta Mass	%Relative	%Total
5214.925	6.57E+007	0.052	7.82	0.000	100.00	80.51
5236.896	8.71E+006	0.052	6.94	21.971	13.25	10.67
5528.974	2.83E+006	0.055	6.45	314.049	4.31	3.47
5237.896	1.98E+006	0.052	6.30	22.971	3.01	2.42
5215.928	1.71E+006	0.052	6.23	1.003	2.60	2.09
5550.954	6.85E+005	0.056	5.84	336.029	1.04	0.84

ZNova 3.0.12 ©2001-2018 Novatia, LLC

[<<] [Top] [ESI Mass Spectrum] [Deconvolution] [View Data] [Log File]

Deconvolution Peak Report:

Mass (Da)	Intensity	Std. Dev.	Score	Delta Mass	%Relative	%Total
5175.915	1.11E+007	0.052	7.04	0.000	100.00	87.20
5197.891	7.55E+005	0.053	5.88	21.976	6.82	5.95
5489.964	3.76E+005	0.055	5.58	314.049	3.40	2.96
5196.928	2.14E+005	0.052	5.33	21.013	1.93	1.68
5490.965	1.44E+005	0.055	5.16	315.050	1.30	1.14
5198.885	1.36E+005	0.052	5.13	22.970	1.22	1.07

ZNova 3.0.12 ©2001-2018 Novatia, LLC

[<<] [Top] [ESI Mass Spectrum] [Deconvolution] [View Data] [Log File]

Deconvolution Peak Report:

Mass (Da)	Intensity	Std. Dev.	Score	Delta Mass	%Relative	%Total
5230.920	1.44E+007	0.052	7.16	0.000	100.00	83.45
5252.892	1.57E+006	0.053	6.20	21.972	10.92	9.11
5544.967	5.47E+005	0.055	5.74	314.047	3.79	3.17
5253.891	3.70E+005	0.053	5.57	22.971	2.57	2.14
5231.926	3.67E+005	0.052	5.57	1.006	2.55	2.13

ZNova 3.0.12 ©2001-2018 Novatia, LLC

[<<] [Top] [ESI Mass Spectrum] [Deconvolution] [View Data] [Log File]

Deconvolution Peak Report:

Mass (Da)	Intensity	Std. Dev.	Score	Delta Mass	%Relative	%Total
5215.921	1.92E+007	0.052	7.28	0.000	100.00	80.83
5237.891	2.77E+006	0.052	6.44	21.970	14.43	11.66
5238.892	7.08E+005	0.052	5.85	22.971	3.69	2.98
5529.967	6.32E+005	0.055	5.80	314.046	3.29	2.66
5259.869	2.24E+005	0.053	5.35	43.948	1.17	0.94
5530.967	2.18E+005	0.055	5.34	315.046	1.14	0.92

ZNova 3.0.12 ©2001-2018 Novatia, LLC

[<<] [Top] [ESI Mass Spectrum] [Deconvolution] [View Data] [Log File]

Deconvolution Peak Report:

Mass (Da)	Intensity	Std. Dev.	Score	Delta Mass	%Relative	%Total
5191.912	1.41E+007	0.052	7.15	0.000	100.00	84.01
5213.878	1.39E+006	0.053	6.14	21.966	9.85	8.28
5214.885	8.21E+005	0.052	5.91	22.973	5.83	4.90
5505.951	4.72E+005	0.055	5.67	314.039	3.36	2.82

ZNova 3.0.12 ©2001-2018 Novatia, LLC

[<<] [Top] [ESI Mass Spectrum] [Deconvolution] [View Data] [Log File]

Deconvolution Peak Report:

Mass (Da)	Intensity	Std. Dev.	Score	Delta Mass	%Relative	%Total
5190.914	1.55E+007	0.052	7.19	0.000	100.00	79.91
5212.885	1.83E+006	0.052	6.26	21.971	11.86	9.47
5504.960	5.85E+005	0.055	5.77	314.046	3.78	3.02
5213.882	5.30E+005	0.052	5.72	22.968	3.43	2.74
4901.865	3.23E+005	0.049	5.51	-289.049	2.09	1.67
5214.888	2.25E+005	0.052	5.35	23.974	1.46	1.16
5505.962	2.16E+005	0.055	5.34	315.048	1.40	1.12
5191.917	1.74E+005	0.052	5.24	1.003	1.13	0.90

ZNova 3.0.12 ©2001-2018 Novatia, LLC

[<<] [Top] [ESI Mass Spectrum] [Deconvolution] [View Data] [Log File]

Deconvolution Peak Report:

Mass (Da)	Intensity	Std. Dev.	Score	Delta Mass	%Relative	%Total
5175.915	1.47E+007	0.052	7.17	0.000	100.00	79.74
5197.887	2.00E+006	0.052	6.30	21.972	13.62	10.86
5198.887	4.77E+005	0.052	5.68	22.972	3.24	2.59
5489.961	4.62E+005	0.055	5.66	314.046	3.14	2.51
4886.860	2.46E+005	0.050	5.39	-289.055	1.67	1.33
5176.919	1.94E+005	0.052	5.29	1.004	1.32	1.05
5490.964	1.84E+005	0.055	5.26	315.049	1.25	1.00
5219.868	1.70E+005	0.052	5.23	43.953	1.16	0.92

ZNova 3.0.12 ©2001-2018 Novatia, LLC

[<<] [Top] [ESI Mass Spectrum] [Deconvolution] [View Data] [Log File]

Deconvolution Peak Report:

Mass (Da)	Intensity	Std. Dev.	Score	Delta Mass	%Relative	%Total
5151.902	1.20E+007	0.052	7.08	0.000	100.00	81.86
5173.879	1.19E+006	0.053	6.07	21.977	9.91	8.11
5174.876	5.76E+005	0.052	5.76	22.974	4.80	3.93
5465.946	4.28E+005	0.055	5.63	314.044	3.57	2.92
4862.848	1.88E+005	0.050	5.28	-289.054	1.57	1.29
5175.880	1.53E+005	0.052	5.19	23.978	1.28	1.05
5466.954	1.23E+005	0.055	5.09	315.052	1.03	0.84

ZNova 3.0.12 ©2001-2018 Novatia, LLC

[<<] [Top] [ESI Mass Spectrum] [Deconvolution] [View Data] [Log File]

Deconvolution Peak Report:

Mass (Da)	Intensity	Std. Dev.	Score	Delta Mass	%Relative	%Total
5195.861	6.66E+008	0.052	8.82	0.000	100.00	85.80
5217.829	5.95E+007	0.052	7.77	21.968	8.94	7.67
5509.908	2.30E+007	0.055	7.36	314.047	3.46	2.97
5196.861	1.18E+007	0.052	7.07	1.000	1.77	1.52
5218.830	8.72E+006	0.052	6.94	22.969	1.31	1.12
4891.810	7.08E+006	0.049	6.85	-304.051	1.06	0.91

ZNova 3.0.12 ©2001-2018 Novatia, LLC

[<<] [Top] [ESI Mass Spectrum] [Deconvolution] [View Data] [Log File]

Deconvolution Peak Report:

Mass (Da)	Intensity	Std. Dev.	Score	Delta Mass	%Relative	%Total
5285.882	1.06E+008	0.053	8.03	0.000	100.00	78.39
5307.849	1.30E+007	0.053	7.11	21.967	12.20	9.57
5379.930	4.67E+006	0.054	6.67	94.048	4.39	3.44
5599.930	3.29E+006	0.056	6.52	314.048	3.10	2.43
5195.861	3.01E+006	0.052	6.48	-90.021	2.83	2.22
5308.849	2.51E+006	0.053	6.40	22.967	2.36	1.85
5600.927	1.45E+006	0.056	6.16	315.045	1.37	1.07
5205.900	1.41E+006	0.052	6.15	-79.982	1.32	1.04

ZNova 3.0.12 ©2001-2018 Novatia, LLC

[<<] [Top] [ESI Mass Spectrum] [Deconvolution] [View Data] [Log File]

Deconvolution Peak Report:

Mass (Da)	Intensity	Std. Dev.	Score	Delta Mass	%Relative	%Total
5279.892	1.81E+007	0.053	7.26	0.000	100.00	72.42
5301.862	2.28E+006	0.053	6.36	21.970	12.59	9.11
5285.891	2.22E+006	0.053	6.35	5.999	12.27	8.88
5195.860	1.27E+006	0.052	6.10	-84.032	6.98	5.06
5593.939	5.23E+005	0.056	5.72	314.047	2.89	2.09
5302.861	4.09E+005	0.053	5.61	22.969	2.26	1.64
5594.941	2.00E+005	0.056	5.30	315.049	1.11	0.80

ZNova 3.0.12 ©2001-2018 Novatia, LLC

[<<] [Top] [ESI Mass Spectrum] [Deconvolution] [View Data] [Log File]

Deconvolution Peak Report:

Mass (Da)	Intensity	Std. Dev.	Score	Delta Mass	%Relative	%Total
5246.870	1.35E+008	0.053	8.13	0.000	100.00	84.59
5268.837	1.64E+007	0.054	7.22	21.967	12.22	10.34
5560.919	4.55E+006	0.056	6.66	314.049	3.38	2.86
5269.840	2.13E+006	0.053	6.33	22.970	1.59	1.34
5270.838	1.38E+006	0.053	6.14	23.968	1.03	0.87

ZNova 3.0.12 ©2001-2018 Novatia, LLC

[<<] [Top] [ESI Mass Spectrum] [Deconvolution] [View Data] [Log File]

Deconvolution Peak Report:

Mass (Da)	Intensity	Std. Dev.	Score	Delta Mass	%Relative	%Total
5311.880	4.39E+007	0.053	7.64	0.000	100.00	71.35
5333.849	7.80E+006	0.053	6.89	21.969	17.79	12.69
5246.867	3.15E+006	0.052	6.50	-65.013	7.19	5.13
5625.930	1.76E+006	0.056	6.24	314.050	4.01	2.86
5334.853	1.19E+006	0.053	6.07	22.973	2.71	1.93
5285.877	6.34E+005	0.053	5.80	-26.003	1.45	1.03
5195.856	6.19E+005	0.054	5.79	-116.024	1.41	1.01
5355.832	6.05E+005	0.055	5.78	43.952	1.38	0.98
5335.851	4.84E+005	0.053	5.68	23.971	1.10	0.79
5626.931	4.79E+005	0.056	5.68	315.051	1.09	0.78
5432.937	4.51E+005	0.054	5.65	121.057	1.03	0.73
5647.900	4.41E+005	0.056	5.64	336.020	1.01	0.72

ZNova 3.0.12 ©2001-2018 Novatia, LLC

[<<] [Top] [ESI Mass Spectrum] [Deconvolution] [View Data] [Log File]

Deconvolution Peak Report:

Mass (Da)	Intensity	Std. Dev.	Score	Delta Mass	%Relative	%Total
5270.880	4.89E+006	0.053	6.69	0.000	100.00	38.67
5246.870	1.37E+006	0.053	6.14	-24.010	28.04	10.84
5452.918	1.06E+006	0.057	6.02	182.038	21.61	8.35
5311.878	1.04E+006	0.053	6.02	40.998	21.27	8.22
5292.854	6.69E+005	0.054	5.83	21.974	13.67	5.29
5326.873	4.76E+005	0.053	5.68	55.993	9.72	3.76
5195.862	4.31E+005	0.052	5.63	-75.018	8.80	3.40
5285.881	4.17E+005	0.054	5.62	15.001	8.52	3.29
5634.938	2.03E+005	0.058	5.31	364.058	4.14	1.60
5269.850	1.91E+005	0.054	5.28	-1.030	3.90	1.51
5453.919	1.65E+005	0.055	5.22	183.039	3.37	1.30
5408.908	1.51E+005	0.054	5.18	138.028	3.08	1.19
5333.871	1.42E+005	0.059	5.15	62.991	2.91	1.13
5584.921	1.41E+005	0.056	5.15	314.041	2.89	1.12
5310.859	1.21E+005	0.055	5.08	39.979	2.48	0.96
5480.877	1.18E+005	0.055	5.07	209.997	2.42	0.93
5474.896	1.17E+005	0.058	5.07	204.016	2.38	0.92
5293.855	1.16E+005	0.053	5.06	22.975	2.36	0.91
5279.891	1.01E+005	0.053	5.01	9.011	2.07	0.80
5513.865	1.01E+005	0.057	5.01	242.985	2.07	0.80
4941.833	7.99E+004	0.050	4.90	-329.047	1.63	0.63
5312.879	7.74E+004	0.054	4.89	41.999	1.58	0.61
5508.891	7.64E+004	0.057	4.88	238.011	1.56	0.60
5271.886	7.01E+004	0.053	4.85	1.006	1.43	0.55
5407.894	6.43E+004	0.055	4.81	137.014	1.31	0.51
5268.871	5.66E+004	0.053	4.75	-2.009	1.16	0.45
5245.866	5.46E+004	0.055	4.74	-25.014	1.12	0.43
5286.884	5.35E+004	0.053	4.73	16.004	1.09	0.42
5663.915	5.00E+004	0.058	4.70	393.035	1.02	0.40
5457.915	4.96E+004	0.055	4.70	187.035	1.01	0.39

ZNova 3.0.12 ©2001-2018 Novatia, LLC

[<<] [Top] [ESI Mass Spectrum] [Deconvolution] [View Data] [Log File]

Deconvolution Peak Report:

Mass (Da)	Intensity	Std. Dev.	Score	Delta Mass	%Relative	%Total
5249.861	1.09E+007	0.053	7.04	0.000	100.00	57.52
5271.833	3.46E+006	0.053	6.54	21.972	31.80	18.29
5272.831	8.41E+005	0.053	5.92	22.970	7.73	4.45
5246.869	8.05E+005	0.053	5.91	-2.992	7.40	4.25
5311.865	5.37E+005	0.054	5.73	62.004	4.94	2.84
5563.907	4.49E+005	0.056	5.65	314.046	4.12	2.37
5293.812	3.61E+005	0.053	5.56	43.951	3.31	1.91
5305.838	3.15E+005	0.053	5.50	55.977	2.90	1.67
5248.874	2.94E+005	0.053	5.47	-0.987	2.70	1.55
5294.808	2.00E+005	0.054	5.30	44.947	1.84	1.06
5270.862	1.85E+005	0.055	5.27	21.001	1.70	0.98
5268.859	1.83E+005	0.054	5.26	18.998	1.68	0.97
5585.885	1.79E+005	0.056	5.25	336.024	1.65	0.95
5286.881	1.14E+005	0.053	5.06	37.020	1.05	0.60
5304.847	1.13E+005	0.053	5.05	54.986	1.04	0.60

ZNova 3.0.12 ©2001-2018 Novatia, LLC

[<<] [Top] [ESI Mass Spectrum] [Deconvolution] [View Data] [Log File]

Deconvolution Peak Report:

Mass (Da)	Intensity	Std. Dev.	Score	Delta Mass	%Relative	%Total
5257.872	1.72E+007	0.053	7.23	0.000	100.00	63.57
5279.845	5.49E+006	0.053	6.74	21.973	31.97	20.32
5571.916	6.65E+005	0.056	5.82	314.044	3.87	2.46
5246.868	6.36E+005	0.053	5.80	-11.004	3.70	2.35
5280.850	6.01E+005	0.053	5.78	22.978	3.50	2.22
5281.845	4.60E+005	0.053	5.66	23.973	2.68	1.71
5301.823	3.51E+005	0.054	5.54	43.951	2.04	1.30
5311.874	3.34E+005	0.053	5.52	54.002	1.95	1.24
5195.864	2.80E+005	0.053	5.45	-62.008	1.63	1.04
5593.895	2.70E+005	0.056	5.43	336.023	1.57	1.00
5249.885	2.01E+005	0.054	5.30	-7.987	1.17	0.74
5285.859	1.92E+005	0.053	5.28	27.987	1.12	0.71
5302.815	1.89E+005	0.053	5.28	44.943	1.10	0.70
5572.921	1.73E+005	0.056	5.24	315.049	1.01	0.64

ZNova 3.0.12 ©2001-2018 Novatia, LLC

[<<] [Top] [ESI Mass Spectrum] [Deconvolution] [View Data] [Log File]
Deconvolution Peak Report:

Mass (Da)	Intensity	Std. Dev.	Score	Delta Mass	%Relative	%Total
5294.893	3.26E+007	0.053	7.51	0.000	100.00	79.51
5316.863	4.10E+006	0.053	6.61	21.970	12.57	9.99
5608.941	1.28E+006	0.056	6.11	314.048	3.92	3.12
5317.862	1.23E+006	0.053	6.09	22.969	3.78	3.00
4981.836	6.90E+005	0.050	5.84	-313.057	2.11	1.68
5246.867	3.78E+005	0.054	5.58	-48.026	1.16	0.92
5609.943	3.74E+005	0.056	5.57	315.050	1.15	0.91
5257.876	3.56E+005	0.053	5.55	-37.017	1.09	0.87

ZNova 3.0.12 ©2001-2018 Novatia, LLC

[<<] [Top] [ESI Mass Spectrum] [Deconvolution] [View Data] [Log File]

Deconvolution Peak Report:

Mass (Da)	Intensity	Std. Dev.	Score	Delta Mass	%Relative	%Total
5255.880	2.19E+007	0.053	7.34	0.000	100.00	77.84
5277.855	2.40E+006	0.053	6.38	21.975	10.96	8.53
5569.928	7.70E+005	0.056	5.89	314.048	3.52	2.74
5294.882	7.35E+005	0.055	5.87	39.002	3.36	2.62
5278.854	6.74E+005	0.053	5.83	22.974	3.08	2.40
4942.824	4.97E+005	0.050	5.70	-313.056	2.27	1.77
5246.872	3.61E+005	0.052	5.56	-9.008	1.65	1.29
5570.932	2.72E+005	0.056	5.43	315.052	1.24	0.97
4966.822	2.62E+005	0.051	5.42	-289.058	1.20	0.93
5195.860	2.60E+005	0.052	5.41	-60.020	1.19	0.92

ZNova 3.0.12 ©2001-2018 Novatia, LLC

[<<] [Top] [ESI Mass Spectrum] [Deconvolution] [View Data] [Log File]

Deconvolution Peak Report:

Mass (Da)	Intensity	Std. Dev.	Score	Delta Mass	%Relative	%Total
5310.888	2.81E+007	0.053	7.45	0.000	100.00	80.67
5332.858	3.68E+006	0.053	6.57	21.970	13.09	10.56
5624.934	1.10E+006	0.056	6.04	314.046	3.90	3.15
5333.859	7.32E+005	0.053	5.86	22.971	2.61	2.10
5625.938	3.46E+005	0.056	5.54	315.050	1.23	0.99
5255.880	3.10E+005	0.053	5.49	-55.008	1.10	0.89
5294.884	2.87E+005	0.054	5.46	-16.004	1.02	0.83
5646.913	2.84E+005	0.056	5.45	336.025	1.01	0.82

ZNova 3.0.12 ©2001-2018 Novatia, LLC

[<<] [Top] [ESI Mass Spectrum] [Deconvolution] [View Data] [Log File]

Deconvolution Peak Report:

Mass (Da)	Intensity	Std. Dev.	Score	Delta Mass	%Relative	%Total
5295.889	2.95E+007	0.053	7.47	0.000	100.00	77.53
5317.859	5.14E+006	0.053	6.71	21.970	17.40	13.49
5318.858	1.10E+006	0.053	6.04	22.969	3.72	2.89
5609.936	9.33E+005	0.056	5.97	314.047	3.16	2.45
5339.830	4.19E+005	0.054	5.62	43.941	1.42	1.10
5311.890	3.54E+005	0.053	5.55	16.001	1.20	0.93
5310.884	3.17E+005	0.054	5.50	14.995	1.07	0.83
5610.940	2.99E+005	0.056	5.48	315.051	1.01	0.78

ZNova 3.0.12 ©2001-2018 Novatia, LLC

[<<] [Top] [ESI Mass Spectrum] [Deconvolution] [View Data] [Log File]

Deconvolution Peak Report:

Mass (Da)	Intensity	Std. Dev.	Score	Delta Mass	%Relative	%Total
5271.876	3.77E+007	0.053	7.58	0.000	100.00	80.25
5293.847	4.26E+006	0.053	6.63	21.971	11.30	9.07
5294.849	1.84E+006	0.053	6.26	22.973	4.86	3.90
5585.920	1.28E+006	0.056	6.11	314.044	3.39	2.72
5295.851	8.23E+005	0.053	5.92	23.975	2.18	1.75
4982.829	6.80E+005	0.050	5.83	-289.047	1.80	1.45
5586.927	4.05E+005	0.056	5.61	315.051	1.07	0.86

ZNova 3.0.12 ©2001-2018 Novatia, LLC

[<<] [Top] [ESI Mass Spectrum] [Deconvolution] [View Data] [Log File]

Deconvolution Peak Report:

Mass (Da)	Intensity	Std. Dev.	Score	Delta Mass	%Relative	%Total
5270.882	3.40E+007	0.053	7.53	0.000	100.00	78.39
5292.853	4.21E+006	0.053	6.62	21.971	12.40	9.72
5271.886	1.10E+006	0.053	6.04	1.004	3.22	2.53
5584.932	1.09E+006	0.056	6.04	314.050	3.20	2.51
5294.854	1.00E+006	0.053	6.00	23.972	2.95	2.31
4981.829	6.41E+005	0.050	5.81	-289.053	1.89	1.48
5293.852	5.51E+005	0.053	5.74	22.970	1.62	1.27
4957.825	4.14E+005	0.050	5.62	-313.057	1.22	0.95
5585.931	3.70E+005	0.056	5.57	315.049	1.09	0.85

ZNova 3.0.12 ©2001-2018 Novatia, LLC

[<<] [Top] [ESI Mass Spectrum] [Deconvolution] [View Data] [Log File]

Deconvolution Peak Report:

Mass (Da)	Intensity	Std. Dev.	Score	Delta Mass	%Relative	%Total
5255.883	2.61E+007	0.053	7.42	0.000	100.00	75.02
5277.855	4.63E+006	0.053	6.67	21.972	17.76	13.33
5278.855	9.82E+005	0.053	5.99	22.972	3.77	2.83
5569.931	9.05E+005	0.056	5.96	314.048	3.47	2.60
4966.832	5.59E+005	0.050	5.75	-289.051	2.14	1.61
5271.880	5.39E+005	0.053	5.73	15.997	2.07	1.55
5270.882	4.99E+005	0.053	5.70	14.999	1.91	1.44
5299.839	2.96E+005	0.053	5.47	43.956	1.14	0.85
5570.934	2.70E+005	0.056	5.43	315.051	1.04	0.78

ZNova 3.0.12 ©2001-2018 Novatia, LLC

[<<] [Top] [ESI Mass Spectrum] [Deconvolution] [View Data] [Log File]

Deconvolution Peak Report:

Mass (Da)	Intensity	Std. Dev.	Score	Delta Mass	%Relative	%Total
5231.870	4.05E+007	0.052	7.61	0.000	100.00	78.33
5253.843	5.54E+006	0.053	6.74	21.973	13.69	10.72
4942.820	1.35E+006	0.050	6.13	-289.050	3.34	2.62
5545.917	1.31E+006	0.056	6.12	314.047	3.23	2.53
5254.841	1.15E+006	0.052	6.06	22.971	2.85	2.23
5255.842	9.67E+005	0.053	5.99	23.972	2.39	1.87
5546.920	4.58E+005	0.055	5.66	315.050	1.13	0.89
4918.812	4.18E+005	0.049	5.62	-313.058	1.03	0.81

ZNova 3.0.12 ©2001-2018 Novatia, LLC

1.4.4 LC UV-Vis chromatograms for controls and reactions

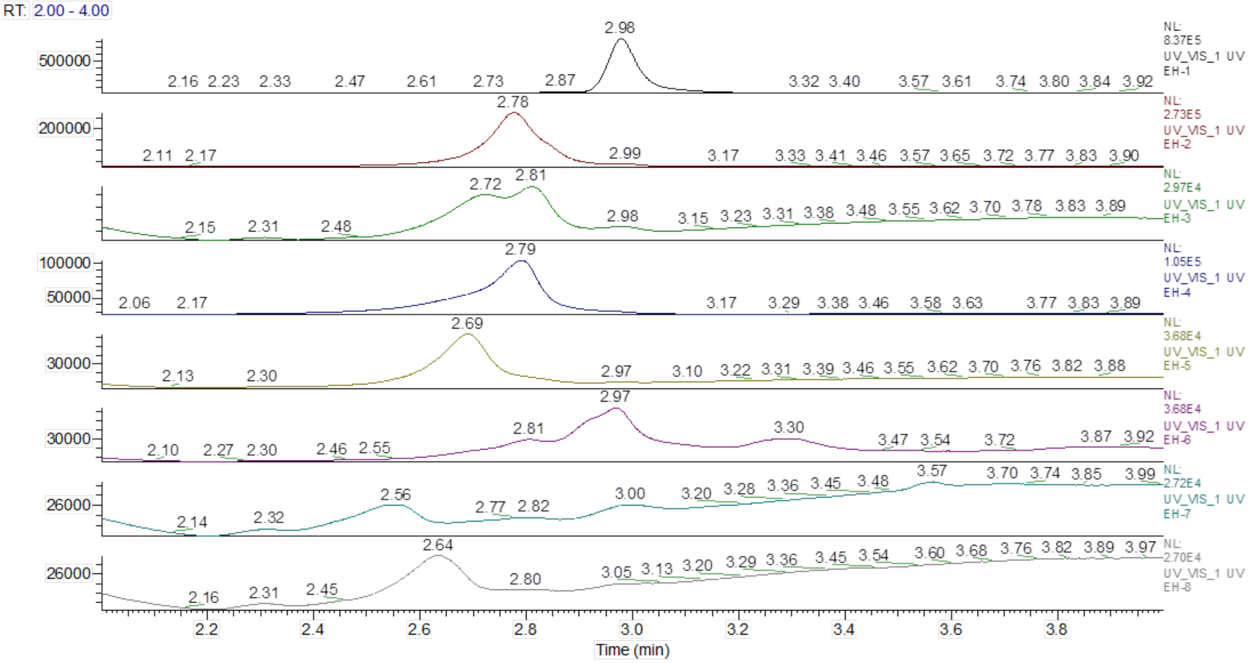

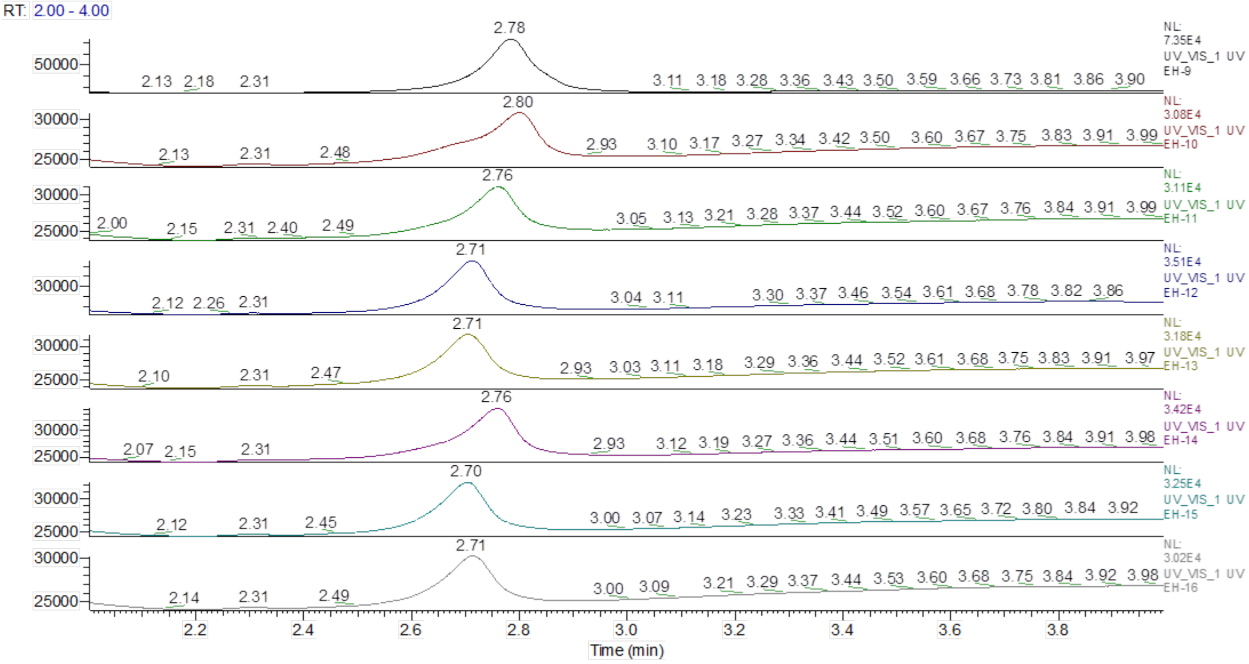

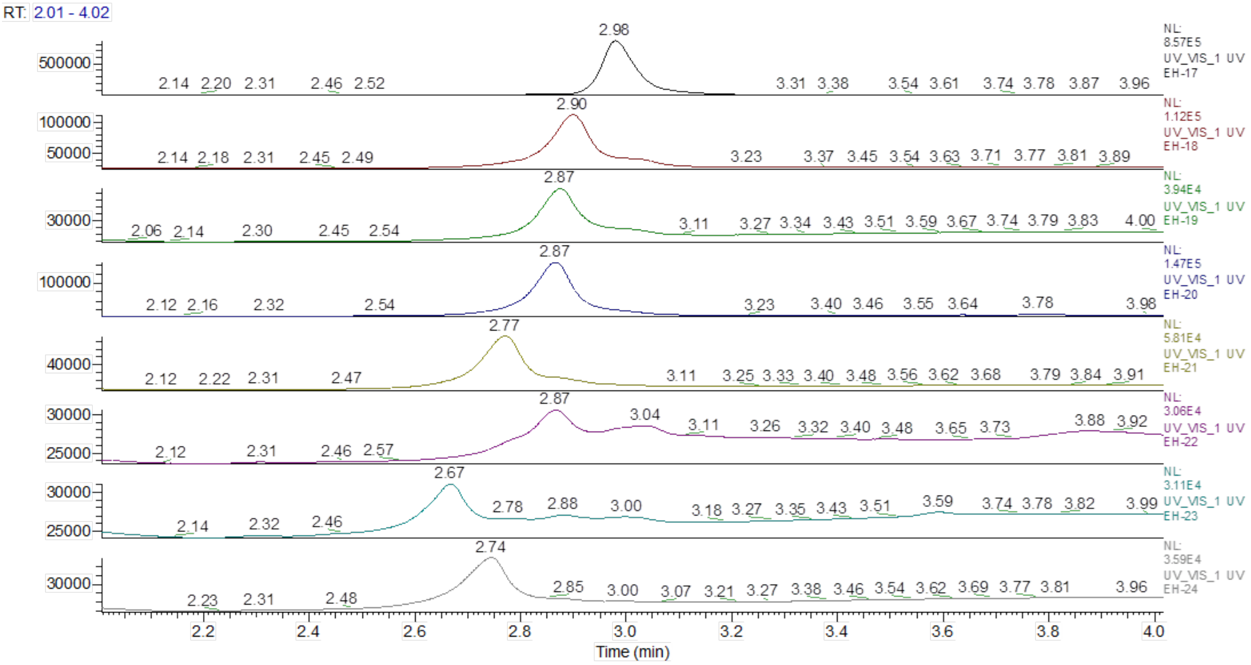

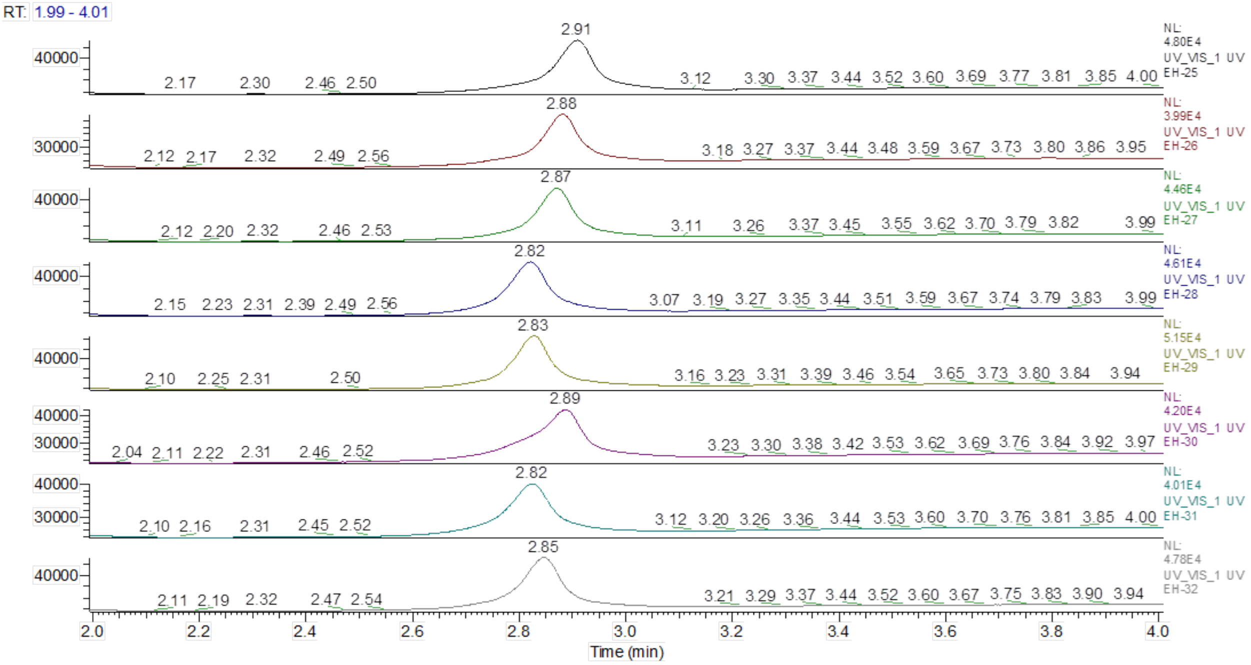

1.5 Statistical Analysis of Guide Population Differences

Data Load-In

To run this notebook from your own R session, be sure to download the supplementary .csv data file to the same directory as this .Rmd notebook file.

```
guideData <- readr::read_csv("guideData.csv", col_types = "ccccncccccnlclnnnnnnnn") %>%
  dplyr::filter(ssds == "ds" | (ssds == "ss" & FWRV == "FW")) %>%
  tibble::add_column(g12 = stringr::str_sub(.$seq_id, start = 11, end = 11), .after = "g1") %>%
  purrr::modify_at(.at = vars(full_comp), factor, levels = c("TRUE", "FALSE"))
guideData
```

```
## # A tibble: 2,946 x 23
##    ssds  target_name target_spec FWRV  prod_size guide_seq   seq_id  g1    g12
##    <chr> <chr>       <chr>       <chr>     <dbl> <chr>       <chr>   <chr> <chr>
##  1 ds    ranTar      30          FW            7 ATCAACTCCA~ TCAACT~ A     T
##  2 ds    ranTar      30          FW            7 CTCAACTCCA~ TCAACT~ C     T
##  3 ds    ranTar      30          FW            7 GTCAACTCCA~ TCAACT~ G     T
##  4 ds    ranTar      30          FW            7 TTCAACTCCA~ TCAACT~ T     T
##  5 ds    ranTar      30          FW            8 ATTCAACTCC~ TTCAAC~ A     A
##  6 ds    ranTar      30          FW            8 CTTCAACTCC~ TTCAAC~ C     A
##  7 ds    ranTar      30          FW            8 GTTCAACTCC~ TTCAAC~ G     A
##  8 ds    ranTar      30          FW            8 TTTCAACTCC~ TTCAAC~ T     A
##  9 ds    ranTar      30          FW            9 AATTCAACTC~ ATTCAA~ A     A
## 10 ds    ranTar      30          FW            9 CATTCAACTC~ ATTCAA~ C     A
## # ... with 2,936 more rows, and 14 more variables: target_seq <chr>, t1 <chr>,
## #   target_gc <dbl>, full_comp <fct>, buffer <chr>, preload <lgl>, ETSSB <dbl>,
## #   rxn_temp_C <dbl>, rxn_time_min <dbl>, act_sd <dbl>, act <dbl>,
## #   act_bp <dbl>, offsense <dbl>, offanti <dbl>
```

Multiple Comparison Analysis for t1 Grouping

Analysis of variance was performed on the *Tt*Ago guide subsets according to both the stranded-ness of the substrate and the identity of the first base at the 3' end of the target sequence (t1), which aligns with the first base at the 5' end of the guide (g1). The g1 identity was used to generate the grouping within each of these subsets.

Following variance analysis of each of these subsets, where variance was statistically significant by the Kruskal-Wallis method, post-hoc multiple comparison pairwise analysis was performed between all g1 groups using the Dunn method.

Only the pairwise relationships between complementary group (where g1 was complementary to t1) and the other three identities of g1 were reported on the plots. Reported p-values were corrected for sampling by the Bonferroni method, and these values were used to generate the star notation found on the plots (where ns is $p > 0.05$; * is $p \leq 0.05$, ** is $p \leq 0.01$; *** is $p \leq 0.001$; and **** is $p \leq 0.0001$).

Data with t1 grouping

Data is grouped by ssds and t1 identity A, C, G, or T. Single-stranded substrate data contains results for only the FW guides.

```
t1Data <- guideData %>%
  split(f = list(.$t1, .$ssds))

t1Data <- do.call(
  rlang::set_names,
  list(
    t1Data,
    nm = map_chr(t1Data, ~ paste0("t1", unique(.x$t1), unique(.x$ssds)))
  )
)
# map(t1Data, head)
```

Kruskal-Wallis

ANOVA is not the best choice as the data are often left or right skewed and not 'normal' because activity cannot be lower than zero or higher than 100%. Instead of ANOVA, Kruskal-Wallis was chosen as a 'ranked' one-way ANOVA which makes no assumptions about the data being 'normal.'

```
t1Kruskal <- t1Data %>%
  purrr::map_dfr(
    ~ rstatix::kruskal_test(
      .x,
      act ~ g1
    ),
    .id = "t1ssds"
  ) %>%
  dplyr::mutate(t1ssds = stringr::str_extract(t1ssds, "(?<=t1)[ACGT]?(s|d)s$")) %>%
  tidyr::separate(t1ssds, 1, into = c("t1", "ssds")) %>%
  dplyr::arrange(desc(ssds), t1)
t1Kruskal
```

t1	ssds	.y.	n	statistic	df	p	method
A	ss	act	243	17.17753	3	6.50e-04	Kruskal-Wallis
C	ss	act	224	49.57162	3	0.00e+00	Kruskal-Wallis
G	ss	act	252	31.82177	3	6.00e-07	Kruskal-Wallis
T	ss	act	264	24.73930	3	1.75e-05	Kruskal-Wallis
A	ds	act	498	20.84533	3	1.13e-04	Kruskal-Wallis
C	ds	act	478	36.48108	3	1.00e-07	Kruskal-Wallis
G	ds	act	467	21.16444	3	9.73e-05	Kruskal-Wallis
T	ds	act	520	55.57907	3	0.00e+00	Kruskal-Wallis

Post-hoc Dunn Test

To determine which median comparisons are statistically significant within each group tested by Kruskal-Wallis.

```
t1Dunn <- t1Data %>%
  purrr::map_dfr(
    ~ rstatix::dunn_test(
      .x,
      act ~ g1,
      p.adjust.method = "bonferroni"
      # p.adjust.method = "holm"
    ),
    .id = "t1ssds"
  ) %>%
  dplyr::mutate(t1ssds = stringr::str_extract(t1ssds, "(?<=t1)[ACGT]?(s|d)s$")) %>%
  tidyr::separate(t1ssds, 1, into = c("t1", "ssds")) %>%
  dplyr::rename(g1A = group1, g1B = group2) %>%
  # dplyr::mutate(grouprev = map_chr(t1, seq_revcomp)) %>%
  # dplyr::relocate(grouprev, .after = t1) %>%
  dplyr::filter(
    map_chr(t1, seq_revcomp) == g1A |
      map_chr(t1, seq_revcomp) == g1B
  ) %>%
  # dplyr::filter(p.adj.signif != "ns") %>%
  dplyr::arrange(desc(ssds), t1) %>%
  dplyr::select(-c(.y., statistic, p))
t1Dunn
```

t1	ssds	g1A	g1B	n1	n2	p.adj	p.adj.signif
A	ss	A	T	60	61	0.0009240	***
A	ss	C	T	61	61	1.0000000	ns
A	ss	G	T	61	61	0.0285890	*
C	ss	A	G	56	56	0.1172141	ns
C	ss	C	G	56	56	0.1463188	ns
C	ss	G	T	56	56	0.0000880	****
G	ss	A	C	63	63	1.0000000	ns
G	ss	C	G	63	63	0.1838627	ns
G	ss	C	T	63	63	0.0002762	***
T	ss	A	C	66	66	0.2124292	ns
T	ss	A	G	66	66	1.0000000	ns
T	ss	A	T	66	66	0.0019598	**
A	ds	A	T	123	125	1.0000000	ns
A	ds	C	T	125	125	0.3839577	ns
A	ds	G	T	125	125	0.0551014	ns
C	ds	A	G	118	120	0.0036876	**
C	ds	C	G	120	120	0.0000099	****
C	ds	G	T	120	120	0.0000001	****
G	ds	A	C	117	116	0.0086942	**
G	ds	C	G	116	117	0.2197121	ns
G	ds	C	T	116	117	0.0000567	****
T	ds	A	C	130	130	0.0000713	****
T	ds	A	G	130	130	1.0000000	ns
T	ds	A	T	130	130	0.0000011	****

The authors note that in panel B, the change from g1G to g1T is not-significant, however, this conclusion is mostly due to the choice to utilize the more conservative p-value correction method (the Bonferroni method). If a less aggressive method such as Holm-Bonferroni is used, the change is significant.

Multiple comparions for g1 grouping between complementary and non-complementary guides

Comparisons of data grouped by t1 provide an understanding of how a change to g1 will affect that a guide with relation to that fully-complementary sequence. However because each group contains a subset where g1 is changed to every other base, in order to make a more complete statistical inference about the effect of g1 on guide suitability, groupings can be created according to g1 such that all guides in which a complementary sequence is altered by changing the first position to a specific base that is not complementary. In other words, for example, in the t1 groupings, changing g1 to a non-complementary T occurs in all groups except t1A. This aids in inference of whether g1 identity has a global effect, or if activity is influenced by both the identity of g1 and the identity of t1.

Generating the data

Function for compling the data with appropriate groups

```
compile_g1Data <- function(data, ..., ssds = NULL) {
  g1N <- list(...)
  if (!length(g1N)) {
    g1N <- purrr::set_names(list("A", "G", "C", "T"))
  } else {
    g1N <- purrr::set_names(g1N)
  }

  if (is.null(ssds)) {
    ssds <- c("ss", "ds")
    FWRV <- c("FW", "RV")
  } else if (!(ssds %in% c("ss", "ds"))) {
    stop("Please choose a strandedness of DNA, as a string.")
  } else if (ssds == "ds") {
    ssds <- c(ssds)
    FWRV <- c("FW", "RV")
  } else if (ssds == "ss") {
    ssds <- c(ssds)
    FWRV <- c("FW")
  }

  abbrevs <- list("A" = "B", "C" = "D", "G" = "H", "T" = "V")
  filters_common <- function(df) {
    df %>%
      filter(ssds == "ds" | (ssds == "ss" & FWRV == "FW")) %>%
      filter(ssds %in% !!ssds, FWRV %in% !!FWRV)
  }

  df <- map_dfr(
    g1N,
    function(N) {
      do.call(
        bind_rows,
        list(
          {
            data %>%
              filters_common() %>%
              filter(full_comp == TRUE & g1 != N) %>%
              mutate(g1 = dplyr::recode(N, !!!abbrevs))
          },
          {
            data %>%
              filters_common() %>%
              filter(full_comp == FALSE & g1 == N)
          }
        )
      )
    },
    .id = "population"
  ) %>%
    modify_at(.at = vars(ssds), factor, levels = c("ss", "ds"), ordered = TRUE) %>%
    modify_at(.at = vars(full_comp), factor, levels = c("TRUE", "FALSE")) %>%
    modify_at(.at = vars(population), factor, levels = c("A", "C", "G", "T"))

  return(df)
}
```

Applying the function

```
g1Data <- compile_g1Data(guideData) %>%
  group_split(ssds, population)

g1Data <- do.call(
  rlang::set_names,
  list(
    g1Data,
    nm = map_chr(g1Data, ~ paste0("g1", unique(.x$population), unique(.x$ssds)))
  )
)
# map(g1Data, head)
```

Kruskal-Wallis

```
g1Kruskal <- g1Data %>%
  purrr::map_dfr(
    ~ rstatix::kruskal_test(
      .x,
      act ~ g1
    ),
    .id = "g1ssds"
  ) %>%
  dplyr::mutate(g1ssds = stringr::str_extract(g1ssds, "(?<=g1)[ACGT]?(s|d)s$")) %>%
  tidyr::separate(g1ssds, 1, into = c("population", "ssds")) %>%
  purrr::modify_at(.at = vars(ssds), factor, levels = c("ss", "ds"), ordered = TRUE) %>%
  dplyr::arrange(ssds, population)
g1Kruskal
```

population	ssds	.y.	n	statistic	df	p	method
A	ss	act	359	16.1039170	1	0.000060	Kruskal-Wallis
C	ss	act	366	3.0240448	1	0.082000	Kruskal-Wallis
G	ss	act	380	0.9430393	1	0.331000	Kruskal-Wallis
T	ss	act	370	43.8622474	1	0.000000	Kruskal-Wallis
A	ds	act	719	15.4897029	1	0.000083	Kruskal-Wallis
C	ds	act	750	37.4302863	1	0.000000	Kruskal-Wallis
G	ds	act	743	0.5094614	1	0.475000	Kruskal-Wallis
T	ds	act	733	73.0846598	1	0.000000	Kruskal-Wallis

Post-hoc Dunn Test

This is a redundant treatment. The final p-value is the same as from Kruskal-Wallis because only two samples are being compared to each other within each g1 subset.

```
g1Dunn <- g1Data %>%
  purrr::map_dfr(
    ~ rstatix::dunn_test(
      .x,
      act ~ g1,
      p.adjust.method = "bonferroni"
      # p.adjust.method = "holm"
    ),
    .id = "g1ssds"
  ) %>%
  dplyr::mutate(g1ssds = stringr::str_extract(g1ssds, "(?<=g1)[ACGT]?(s|d)s$")) %>%
  tidyr::separate(g1ssds, 1, into = c("population", "ssds")) %>%
  dplyr::rename(g1A = group1, g1B = group2) %>%
  dplyr::arrange(desc(ssds), population) %>%
  dplyr::select(-c(.y., statistic, p))
g1Dunn
```

population	ssds	g1A	g1B	n1	n2	p.adj	p.adj.signif
A	ss	A	B	179	180	0.0000600	****
C	ss	C	D	183	183	0.0820386	ns
G	ss	G	H	190	190	0.3314974	ns
T	ss	T	V	185	185	0.0000000	****
A	ds	A	B	358	361	0.0000830	****
C	ds	C	D	375	375	0.0000000	****
G	ds	G	H	372	371	0.4753721	ns
T	ds	T	V	367	366	0.0000000	****

Purine to pyrimidine comparison

As g1C and g1T exhibit the highest activty, a truer comparison would be to eliminate the g1T guides from the comparison subset when considering g1C guides, and vice versa. In other words, the case of g1C and g1T should be compared against the same g1R subset.

Function for compling the data with appropriate groups

```
compile_g1RYData <- function(data, ..., ssds = NULL) {
  g1N <- list(...)
  if (!length(g1N)) {
    g1N <- purrr::set_names(list("C", "T"))
  } else {
    g1N <- purrr::set_names(g1N)
  }

  if (is.null(ssds)) {
    ssds <- c("ss", "ds")
    FWRV <- c("FW", "RV")
  } else if (!(ssds %in% c("ss", "ds"))) {
    stop("Please choose a strandedness of DNA, as a string.")
  } else if (ssds == "ds") {
    ssds <- c(ssds)
    FWRV <- c("FW", "RV")
  } else if (ssds == "ss") {
    ssds <- c(ssds)
    FWRV <- c("FW")
  }

  filters_common <- function(df) {
    df %>%
      filter(ssds == "ds" | (ssds == "ss" & FWRV == "FW")) %>%
      filter(ssds %in% !!ssds, FWRV %in% !!FWRV)
  }

  df <- map_dfr(
    g1N,
    function(N) {
      do.call(
        bind_rows,
        list(
          {
            data %>%
              filters_common() %>%
              filter(full_comp == TRUE & !(g1 %in% g1N)) %>%
              # mutate(g1 = dplyr::recode(N, !!!abbrevs))
              mutate(g1 = "R")
          },
          {
            data %>%
              filters_common() %>%
              filter(full_comp == FALSE & g1 == N & t1 != switch(N, "C" = "A", "T" = "G"))
          }
        )
      )
    },
    .id = "population"
  ) %>%
    modify_at(.at = vars(ssds), factor, levels = c("ss", "ds"), ordered = TRUE) %>%
    modify_at(.at = vars(full_comp), factor, levels = c("TRUE", "FALSE")) %>%
    modify_at(.at = vars(population), factor, levels = c("C", "T"))

  return(df)
}
```

Applying the function

```r
g1RYData <- compile_g1RYData(guideData) %>%
  group_split(ssds, population)

g1RYData <- do.call(
  rlang::set_names,
  list(
    g1RYData,
    nm = map_chr(g1RYData, ~ paste0("g1", unique(.x$population), unique(.x$ssds)))
  )
)

# map(g1RYData, head)
```

Kruskal-Wallis

```r
g1RYKruskal <- g1RYData %>%
  purrr::map_dfr(
    ~ rstatix::kruskal_test(
      .x,
      act ~ g1
    ),
    .id = "g1ssds"
  ) %>%
  dplyr::mutate(g1ssds = stringr::str_extract(g1ssds, "(?<=g1)[ACGT]?(s|d)s$")) %>%
  tidyr::separate(g1ssds, 1, into = c("population", "ssds")) %>%
  purrr::modify_at(.at = vars(ssds), factor, levels = c("ss", "ds"), ordered = TRUE) %>%
  dplyr::arrange(ssds, population)
g1RYKruskal
```

population	ssds	.y.	n	statistic	df	p	method
C	ss	act	244	9.494569	1	2.06e-03	Kruskal-Wallis
T	ss	act	244	29.265530	1	1.00e-07	Kruskal-Wallis
C	ds	act	500	41.586303	1	0.00e+00	Kruskal-Wallis
T	ds	act	500	55.914136	1	0.00e+00	Kruskal-Wallis

Post-hoc Dunn Test

Again , this is a redundant treatment. The final p-value is the same as from Kruskal-Wallis because only two samples are being compared to each other within each g1 subset.

```
g1RYDunn <- g1RYData %>%
  purrr::map_dfr(
    ~ rstatix::dunn_test(
      .x,
      act ~ g1,
      p.adjust.method = "bonferroni"
      # p.adjust.method = "holm"
    ),
    .id = "g1ssds"
  ) %>%
  dplyr::mutate(g1ssds = stringr::str_extract(g1ssds, "(?<=g1)[ACGT]?(s|d)s$")) %>%
  tidyr::separate(g1ssds, 1, into = c("population", "ssds")) %>%
  dplyr::rename(g1A = group1, g1B = group2) %>%
  dplyr::arrange(desc(ssds), population) %>%
  dplyr::select(-c(.y., statistic, p))
g1RYDunn
```

population	ssds	g1A	g1B	n1	n2	p.adj	p.adj.signif
C	ss	C	R	122	122	0.0020608	**
T	ss	R	T	122	122	0.0000001	****
C	ds	C	R	250	250	0.0000000	****
T	ds	R	T	250	250	0.0000000	****

Multiple comparions for g12 grouping

Data with g12 grouping

Data is grouped by ssds and t1 identity A, C, G, or T. Single-stranded substrate data contains results for only the FW guides.

```
g12Data <- guideData %>%
  split(f = list(.$full_comp))

g12Data <- do.call(
  rlang::set_names,
  list(
    g12Data,
    nm = map_chr(g12Data, ~ paste0(unique(.$full_comp)))
  )
)
# map(g12Data, head)
```

Kruskal-Wallis

ANOVA is not the best choice as the data are often left or right skewed and not 'normal' because activity cannot be lower than zero or higher than 100%. Instead of ANOVA, Kruskal-Wallis was chosen as a 'ranked' one-way ANOVA which makes no assumptions about the data being 'normal.'

For all guides

```
g12Kruskal <- g12Data %>%
  purrr::map_dfr(
    ~ rstatix::kruskal_test(
      .x,
      act ~ g12
    ),
    .id = "full_comp"
  ) %>%
  # dplyr::mutate(g12ssds = stringr::str_extract(g12ssds, "(?<=g12)[ACGT]?(s|d)s$")) %>%
  # tidyr::separate(g12ssds, 1, into = c("g12", "full_comp")) %>%
  dplyr::arrange(desc(full_comp))
g12Kruskal
```

full_comp	.y.	n	statistic	df	p	method
TRUE	act	737	47.16440	3	0	Kruskal-Wallis
FALSE	act	2209	82.76808	3	0	Kruskal-Wallis

For g1 = R

```
g12g1RKruskal <- g12Data %>% map(filter, g1 %in% c("A", "G")) %>%
  purrr::map_dfr(
    ~ rstatix::kruskal_test(
      .x,
      act ~ g12
    ),
    .id = "full_comp"
  ) %>%
  # dplyr::mutate(g12ssds = stringr::str_extract(g12ssds, "(?<=g12)[ACGT]?(s|d)s$")) %>%
  # tidyr::separate(g12ssds, 1, into = c("g12", "full_comp")) %>%
  dplyr::arrange(desc(full_comp))
g12g1RKruskal
```

full_comp	.y.	n	statistic	df	p	method
TRUE	act	372	34.72535	3	1e-07	Kruskal-Wallis
FALSE	act	1099	53.20422	3	0e+00	Kruskal-Wallis

For g1 = Y

```
g12g1YKruskal <- g12Data %>% map(filter, g1 %in% c("C", "T")) %>%
  purrr::map_dfr(
    ~ rstatix::kruskal_test(
      .x,
      act ~ g12
    ),
    .id = "full_comp"
  ) %>%
  # dplyr::mutate(g12ssds = stringr::str_extract(g12ssds, "(?<=g12)[ACGT]?(s|d)s$")) %>%
  # tidyr::separate(g12ssds, 1, into = c("g12", "full_comp")) %>%
  dplyr::arrange(desc(full_comp))
g12g1YKruskal
```

full_comp	.y.	n	statistic	df	p	method
TRUE	act	365	26.46015	3	7.6e-06	Kruskal-Wallis
FALSE	act	1110	40.30725	3	0.0e+00	Kruskal-Wallis

For g1 = Y & ssds = ss

```
g12g1YssKruskal <- g12Data %>% map(filter, g1 %in% c("C", "T") & ssds == "ss") %>%
  purrr::map_dfr(
    ~ rstatix::kruskal_test(
      .x,
      act ~ g12
    ),
    .id = "full_comp"
  ) %>%
  # dplyr::mutate(g12ssds = stringr::str_extract(g12ssds, "(?<=g12)[ACGT]?(s|d)s$")) %>%
  # tidyr::separate(g12ssds, 1, into = c("g12", "full_comp")) %>%
  dplyr::arrange(desc(full_comp))
g12g1YssKruskal
```

full_comp	.y.	n	statistic	df	p	method
TRUE	act	124	25.95295	3	9.8e-06	Kruskal-Wallis
FALSE	act	368	66.88460	3	0.0e+00	Kruskal-Wallis

Post-hoc Dunn Test

To determine which median comparisons are statistically significant within each group tested by Kruskal-Wallis.

For all guides

```
g12Dunn <- g12Data %>%
  purrr::map_dfr(
    ~ rstatix::dunn_test(
      .x,
      act ~ g12,
      p.adjust.method = "bonferroni"
      # p.adjust.method = "holm"
    ),
    .id = "full_comp"
  ) %>%
  # dplyr::mutate(g12ssds = stringr::str_extract(g12ssds, "(?<=g12)[ACGT]?(s|d)s$")) %>%
  # tidyr::separate(g12ssds, 1, into = c("g12", "ssds")) %>%
  dplyr::rename(g12A = group1, g12B = group2) %>%
  # dplyr::mutate(grouprev = map_chr(g12, seq_revcomp)) %>%
  # dplyr::relocate(grouprev, .after = g12) %>%
  dplyr::arrange(desc(full_comp)) %>%
  dplyr::select(-c(.y., statistic, p))
g12Dunn
```

full_comp	g12A	g12B	n1	n2	p.adj	p.adj.signif
TRUE	A	C	187	190	0.0000001	****
TRUE	A	G	187	173	0.0000008	****
TRUE	A	T	187	187	0.0000000	****
TRUE	C	G	190	173	1.0000000	ns
TRUE	C	T	190	187	1.0000000	ns
TRUE	G	T	173	187	1.0000000	ns
FALSE	A	C	559	569	0.0000000	****
FALSE	A	G	559	519	0.0000002	****
FALSE	A	T	559	562	0.0000000	****
FALSE	C	G	569	519	1.0000000	ns
FALSE	C	T	569	562	0.4478586	ns
FALSE	G	T	519	562	0.0195676	*

For g1 = R

```
g12g1RDunn <- g12Data %>% map(filter, g1 %in% c("A", "G")) %>%
  purrr::map_dfr(
    ~ rstatix::dunn_test(
      .x,
      act ~ g12,
      p.adjust.method = "bonferroni"
      # p.adjust.method = "holm"
    ),
    .id = "full_comp"
  ) %>%
  # dplyr::mutate(g12ssds = stringr::str_extract(g12ssds, "(?<=g12)[ACGT]?(s|d)s$")) %>%
  # tidyr::separate(g12ssds, 1, into = c("g12", "ssds")) %>%
  dplyr::rename(g12A = group1, g12B = group2) %>%
  # dplyr::mutate(grouprev = map_chr(g12, seq_revcomp)) %>%
  # dplyr::relocate(grouprev, .after = g12) %>%
  dplyr::arrange(desc(full_comp)) %>%
  dplyr::select(-c(.y., statistic, p))
g12g1RDunn
```

full_comp	g12A	g12B	n1	n2	p.adj	p.adj.signif
TRUE	A	C	95	102	0.0000013	****
TRUE	A	G	95	96	0.0000070	****
TRUE	A	T	95	79	0.1310275	ns
TRUE	C	G	102	96	1.0000000	ns
TRUE	C	T	102	79	0.0553847	ns
TRUE	G	T	96	79	0.1179224	ns
FALSE	A	C	277	277	0.0000007	****
FALSE	A	G	277	250	0.0001442	***
FALSE	A	T	277	295	0.0000000	****
FALSE	C	G	277	250	1.0000000	ns
FALSE	C	T	277	295	0.6515364	ns
FALSE	G	T	250	295	0.0693352	ns

For g1 = Y

```
g12g1YDunn <- g12Data %>% map(filter, g1 %in% c("C", "T")) %>%
  purrr::map_dfr(
    ~ rstatix::dunn_test(
      .x,
      act ~ g12,
      p.adjust.method = "bonferroni"
      # p.adjust.method = "holm"
    ),
    .id = "full_comp"
  ) %>%
  # dplyr::mutate(g12ssds = stringr::str_extract(g12ssds, "(?<=g12)[ACGT]?(s|d)s$")) %>%
  # tidyr::separate(g12ssds, 1, into = c("g12", "ssds")) %>%
  dplyr::rename(g12A = group1, g12B = group2) %>%
  # dplyr::mutate(grouprev = map_chr(g12, seq_revcomp)) %>%
  # dplyr::relocate(grouprev, .after = g12) %>%
  dplyr::arrange(desc(full_comp)) %>%
  dplyr::select(-c(.y., statistic, p))
g12g1YDunn
```

full_comp	g12A	g12B	n1	n2	p.adj	p.adj.signif
TRUE	A	C	92	88	0.0137550	*
TRUE	A	G	92	77	0.0377365	*
TRUE	A	T	92	108	0.0000018	****
TRUE	C	G	88	77	1.0000000	ns
TRUE	C	T	88	108	0.3508895	ns
TRUE	G	T	77	108	0.2476025	ns
FALSE	A	C	282	292	0.0000133	****
FALSE	A	G	282	269	0.0005450	***
FALSE	A	T	282	267	0.0000000	****
FALSE	C	G	292	269	1.0000000	ns
FALSE	C	T	292	267	0.9946995	ns
FALSE	G	T	269	267	0.2301472	ns

For g1 = Y & ssds = ss

```
g12g1YssDunn <- g12Data %>% map(filter, g1 %in% c("C", "T") & ssds == "ss") %>%
  purrr::map_dfr(
    ~ rstatix::dunn_test(
      .x,
      act ~ g12,
      p.adjust.method = "bonferroni"
      # p.adjust.method = "holm"
    ),
    .id = "full_comp"
  ) %>%
  # dplyr::mutate(g12ssds = stringr::str_extract(g12ssds, "(?<=g12)[ACGT]?(s|d)s$")) %>%
  # tidyr::separate(g12ssds, 1, into = c("g12", "ssds")) %>%
  dplyr::rename(g12A = group1, g12B = group2) %>%
  # dplyr::mutate(grouprev = map_chr(g12, seq_revcomp)) %>%
  # dplyr::relocate(grouprev, .after = g12) %>%
  dplyr::arrange(desc(full_comp)) %>%
  dplyr::select(-c(.y., statistic, p))
g12g1YssDunn
```

full_comp	g12A	g12B	n1	n2	p.adj	p.adj.signif
TRUE	A	C	32	31	0.0001155	***
TRUE	A	G	32	25	0.0005172	***
TRUE	A	T	32	36	0.0001825	***
TRUE	C	G	31	25	1.0000000	ns
TRUE	C	T	31	36	1.0000000	ns
TRUE	G	T	25	36	1.0000000	ns
FALSE	A	C	96	101	0.0000000	****
FALSE	A	G	96	83	0.0000000	****
FALSE	A	T	96	88	0.0000005	****
FALSE	C	G	101	83	1.0000000	ns
FALSE	C	T	101	88	0.8283618	ns
FALSE	G	T	83	88	0.5938183	ns

2 Supplementary Figures and Tables

2.1 Supplementary Figures

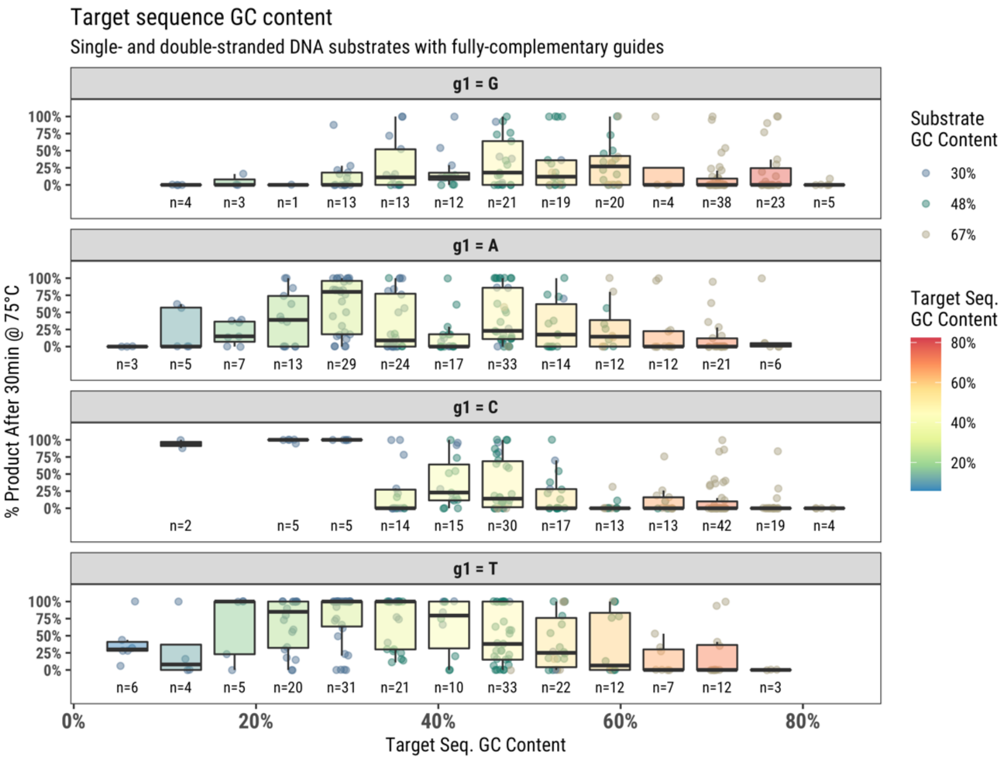

Supplementary Figure 1. Some sequence bias exists due to the overall GC content of the substrates used. For example guides starting with A and T are overrepresented in the population of guides targeting the low GC substrate, and likewise for the high GC substrate for guides starting with G and C. This is unavoidable, but should be noted when considering the extremities.
